# Sustainable Liquid-Phase Exfoliation of Layered Materials with Nontoxic Polarclean Solvent

**DOI:** 10.1021/acssuschemeng.0c04191

**Published:** 2020-12-14

**Authors:** Valentina Paolucci, Gianluca D’Olimpio, Luca Lozzi, Antonio M. Mio, Luca Ottaviano, Michele Nardone, Giuseppe Nicotra, Patrice Le-Cornec, Carlo Cantalini, Antonio Politano

**Affiliations:** † Department of Industrial and Information Engineering and Economics, University of L’Aquila, via G. Gronchi 18, I-67100 L’Aquila, Italy; ‡ Department of Physical and Chemical Sciences, University of L’Aquila, via Vetoio, 67100 L’Aquila, Italy; § CNR-IMM Istituto per la Microelettronica e Microsistemi, VIII strada 5, I-95121 Catania, Italy; ∥ Solvay Novecare, 93308 Auberviliers, France

**Keywords:** Polarclean, green chemistry, layered materials, liquid-phase exfoliation

## Abstract

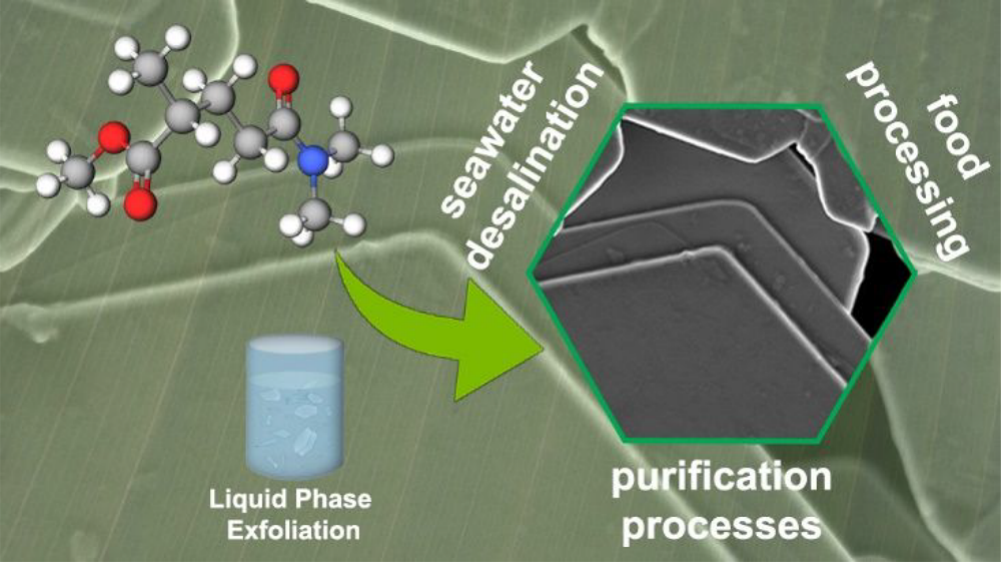

Liquid-phase exfoliation is the most suitable platform for large-scale production of two-dimensional materials. One of the main open challenges is related to the quest of green and bioderived solvents to replace state-of-the-art dispersion media, which suffer several toxicity issues. Here, we demonstrate the suitability of methyl-5-(dimethylamino)-2-methyl-5-oxopentanoate (Rhodiasolv Polarclean) for sonication-assisted liquid-phase exfoliation of layered materials for the case-study examples of WS_2_, MoS_2_, and graphene. We performed a direct comparison, in the same processing conditions, with liquid-phase exfoliation using *N*-methyl-2-pyrrolidone (NMP) solvent. The amount of few-layer flakes (with thickness <5 nm) obtained with Polarclean is increased by ∼350% with respect to the case of liquid-phase exfoliation using NMP, maintaining comparable values of the average lateral size, which even reaches ∼10 μm for the case of graphene produced by exfoliation in Polarclean, and of the yield (∼40%). Correspondingly, the density of defects is reduced by 1 order of magnitude by Polarclean-assisted exfoliation, as evidenced by the *I*(D)/*I*(G) ratio in Raman spectra of graphene as low as 0.07 ± 0.01. Considering the various advantages of Polarclean over state-of-the-art solvents, including the absence of toxicity and its biodegradability, the validation of superior performances of Polarclean in liquid-phase exfoliation paves the way for sustainable large-scale production of nanosheets of layered materials and for extending their use in application fields to date inhibited by toxicity of solvents (e.g., agri-food industry and desalination), with a subsequent superb impact on the commercial potential of their technological applications.

## Abstract 2

Sustainable liquid-phase exfoliation of 2D materials with nontoxic Polarclean solvent for application in agri-food, purification, and desalination processes.

## Introduction

The advent of two-dimensional (2D) materials had a ground-breaking impact on science and technology,^1−13^ due to their peculiar properties with high application capabilities in different fields, such as energy storage,^14−22^ catalysis,^23−27^ optoelectronic devices,^28−31^ and gas sensing.^32−34^ A key point for the technological exploitation of 2D materials is represented by their large-scale production, which still remains challenging.^35−37^ Actually, since the isolation of graphene,^38,39^ fundamental studies on 2D materials were carried out mostly on micrometric flakes mechanically exfoliated from parental bulk crystals^40^ (top-down approach) or on ultrathin layers grown by chemical vapor deposition^41,42^ (bottom-up approach). While mechanical exfoliation suffers from nonscalable processes with scarce reproducibility,^40^ chemical vapor deposition requires specific substrates enabling epitaxial growth,^43−46^ with subsequent problems related to the etching of 2D sheets from the substrate^47^ resulting in flakes with degraded crystalline quality with a high amount of defects and metallic impurities^48^ and/or polymer residuals from the transfer process altering the physicochemical properties of transferred flakes of 2D materials.^49^ The removal of the substrate is a challenging issue also for the preparation of graphene by Si sublimation from SiC substrate.^50^

The most viable tool for large-scale production of few layers of 2D materials is represented by liquid-phase exfoliation (LPE),^37,51−58^ which affords high-quality dispersions of 2D materials, exfoliated from their bulk counterparts and dispersed in solvents enabling further processing.^37,51−54^ Definitely, a suitable solvent for LPE should minimize the energy input required to overcome the van der Waals forces for effective sheet separation.^51−54,58^ This corresponds to the minimization of the enthalpy of mixing per unit volume Δ*H*/*V*, which, in turn, is connected to the Helmholtz free energy of solvent (*F*_solv_) and the Helmholtz free energy of layered materials (*F*_layered_), the thickness of the flakes (*T*_layered_), and the volume fraction (φ):^53,59^
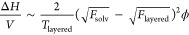
1with
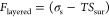
2where σ_s_ is the surface energy and *S*_sur_ the surface entropy.

Therefore, matching surface tensions of solvent and layered materials is crucial to achieve an efficient LPE. However, another critical issue is related to the dispersibility of flakes and solvent, which depends on the specific molecular interactions between the solvent and the solute, which are accounted by considering the Hansen solubility parameters, corresponding to dispersion forces (δ_d_), polar interactions (δ_P_), and hydrogen bonding (δ_H_), respectively. Whenever δ_d_, δ_P_, and δ_H_ of the solvent match the corresponding values for the solute, the energy cost associated with the dispersion is minimized (see the Supporting Information, Section S1, for a more detailed theoretical model for LPE).^51^ For the specific cases of graphene and transition-metal dichalcogenides, *N*-methyl-2-pyrrolidone (NMP) and *N*,*N*-dimethylformamide (DMF) are the most diffusely used solvents,^37^ due to their values of surface tension and Hansen solubility parameters (reported in Table 1) well matching with surface energy and Hansen solubility parameters of graphite and other layered materials (Supporting Information, Table S1). Nevertheless, recently, both NMP and DMF have been placed on the list of Substances of Very High Concern (SVHC),^60^ which is the first step for introducing restrictions over the use of substances or their import to Europe, according to the European REACH regulation.^61^ Similar concerns have been recently raised in the USA for both solvents.^62,63^ In particular, NMP has been already classified as a reproductive toxin,^64^ mainly owing to its amide functionalities.

**Table 1:** Surface Tension and Hansen Solubility Parameters for Polarclean, NMP, DMF, IPA, TEA, and Urea

	surface tension	Hansen solubility parameters		
	σ_S_ [mN m^–1^]	δ_d_ [MPa^1/2^]	δ_p_ [MPa^1/2^]	δ_H_ [MPa^1/2^]
Polarclean	38^82^	15.8^89^	10.7^89^	9.2^89^
NMP	40.1^52^	18.0^59^	12.3^59^	7.2^59^
DMF	37.1^52^	17.4^59^	13.7^59^	11.3^59^
IPA	21.7^52^	15.8^59^	6.1^59^	16.4^59^
TEA	45.9^77^	17.3^104^	7.6^104^	21.0^104^
urea 30% in H_2_O	74.0^105^	17.0^106^	16.7^106^	38.0^106^

Therefore, it is becoming mandatory to search for a *green* alternative to these traditional aprotic solvents.

Volatile organic compounds (VOCs) represent natural candidates as solvents for solution processing, but the exfoliation yield is typically halved,^65^ so that usually the transfer of flakes of 2D materials from a suspension in NMP is required.^66^ In addition, many VOCs have low flash temperatures (13 °C for ethanol; 12 °C for isopropyl alcohol, IPA, etc.), with subsequent concerns for safety for industrial usage.

Another possibility is constituted by LPE in aqueous media using surfactants.^67^ However, residuals of surfactants usually degrade the quality of 2D materials. This drawback is especially relevant for their usage in electronic devices, due to the insulating nature of surfactants, and, moreover, in nanocomposites.^68^

Electrochemical exfoliation (both anodic and cathodic) in aqueous electrolytes has emerged as a novel platform for the production of 2D materials.^62^ However, for bulk semiconductors or insulators, electrochemical exfoliation is unsuccessful in breaking the interlayer van der Waals forces without including a conducting additive.^69^ Moreover, reaching the monolayer regime through electrochemical exfoliation of bulk materials remains a severe hurdle.^70^ Another problem is related to the unconventional operational electrochemical conditions, which imply the occurrence of oxygen and hydrogen evolution stimulated by electrochemical polarization.^71^ Finally, electrochemical exfoliation in aqueous electrolytes usually provides flakes of 2D materials with a high amount of defects.^62,72^

Recently, triethanolamine (TEA)^73^ and urea aqueous solutions^74^ have been proposed as *green* alternative media for LPE of graphene and other layered materials. Regarding TEA, notwithstanding the good results in terms of flake microstructure and dispersion stability, issues related to the yield of the process and, mostly, to chemical modification of flakes induced by possible functionalization^75,76^ during the process are still open. In addition, its very high dynamic viscosity (605.9 cP at *T* = 25 °C^77^) precludes the use of such dispersions for inkjet printing of 2D material-based inks, for which the viscosity range is recommended to be 1–10 cP.^78^ On the other hand, aqueous dispersions of urea have shown encouraging results for graphite exfoliation, obtaining high-quality flakes. However, the low yield of the process (2.4%), evidently related to the significant difference in the surface energy (see Table 1 and the Supporting Information, Section S1), makes urea inappropriate for scalability.

So far, dihydrolevoglucosenone (Cyrene, CAS: 53716-82-8) has been proposed as a green solvent to obtain graphene dispersions.^79^ Cyrene has small, albeit not negligible, values of acute toxicity (LD50) and aquatic toxicity (EC50) of >2000 mg kg and >100 mg L, respectively. Accordingly, its use for many applications of 2D materials, including the production of drinking water through seawater desalination^80^ or other agri-food applications,^81^ is inadvisable. Moreover, the high dynamic viscosity of Cyrene (14.5 cP at *T* = 20 °C) also hinders its use for inkjet printing of 2D material-based inks

Evidently, state-of-the-art methodologies based on common solvents inevitably hamper the long-standing expansion and sustainability of the 2D material-based industry, and concurrently, existing alternatives are far from being mature for mass production of 2D materials. Therefore, the identification of a processing solvent combining (i) efficient LPE and (ii) sustainability remains an open challenge.

**Figure 1:**
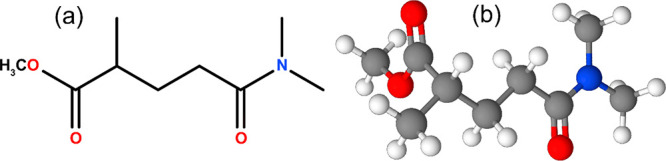
(a) Plain and (b) ball-and-stick representations of the atomic structure of methyl 5-(dimethylamino)-2-methyl-5-oxopentanoate (Polarclean).

Here, we assess the performance of methyl-5-(dimethylamino)-2-methyl-5-oxopentanoate (Rhodiasolv Polarclean, CAS: 1174627-68-9) as a polar solvent^82^ for sonication-assisted LPE of layered materials. Polarclean (C_9_H_17_NO_3_, Figure 1) has no detectable toxicity for doses as high as 1000 mg/(kg day); its water solubility is higher than 490 g/L at *T* = 24 °C, and it is biodegradable and not mutagenic.^83^ Remarkably, Polarclean has a flash point of 160 °C at ambient pressure.^83^ Accordingly, it is safer than many oxygenated solvents, such as VOCs. Besides exfoliation yields and environmental/safety issues, validating a new solvent for 2D material exfoliation means keeping suspension and solvents in use, according to a circular-economy chain-process approach. Notwithstanding, microfiltration^84−86^ represents an interesting route for reuse/recovery of a large variety of exhausted solvents like DMP, NMP, and Polarclean, which increases both the concentrations of the dispersion and the regenerated solvent’s purity; only Polarclean demonstrates large potentials to be reused in downstream production processes. Currently, Polarclean is mostly used for solubilization of agrochemicals, as well as for crop protection and animal nutrition.^87^ Recently, the use of Polarclean has been extended to the production of polymeric membranes for ultrafiltration^88^ and water desalination for drinking water production,^89−92^ the synthesis of biobased aliphatic polyurethanes,^93^ dimerization of abietic acid,^63^ and copper-catalyzed azide–alkyne cycloaddition.^94^ Its dynamic viscosity (9.78 cP at *T* = 23 °C^83^) makes Polarclean an ideal candidate for inkjet printing of 2D material-based devices, for which the low dynamical viscosity of state-of-the-art solvents DMF and NMP (<2 cP) jeopardizes the jetting performance.^95^

Here, we validate the use of Polarclean as the solvent for sonication-assisted LPE of layered materials. Specifically, by adopting as case-study examples WS_2_, MoS_2_, and graphene, we demonstrate that Polarclean outperforms NMP (in the same processing conditions) by producing dispersions of nanosheets with an amount of few-layer flakes (with thickness <5 nm) increased by 350% and comparable values of the average lateral size of flakes. Moreover, the density of defects is reduced by an order of magnitude by exfoliation in Polarclean, as evinced by the *I*(D)/*I*(G) ratio in Raman spectra of graphene as low as 0.07 ± 0.01. Our results indicate that Polarclean represents a unique green candidate solvent for large-scale and scalable production of functional inks based on 2D materials, which naturally enables expanding the use of 2D materials in several application fields, for which state-of-the-art solvents have represented so far serious obstacles, owing to their toxicity. In particular, we mention (i) seawater desalination for production of drinking water;^96^ (ii) concentration of fruit juices,^97^ volatile aroma compounds,^98^ and whey proteins;^99^ (iii) separation of azeotropic mixtures;^100^ (iv) purification processes from fermentation broth;^101^ and (v) recovery of minerals from seawater^102^ and salty lakes.^103^

## Results and Discussion

In Table 1, the value of surface tension and the Hansen solubility parameters of Polarclean are reported and compared with those of other common solvents (NMP, DMF, IPA). The surface tension of Polarclean is comparable with its values for NMP and DMF, while the surface tension in IPA is lower by ∼40%.

The efficiency of Polarclean for obtaining stable and high-yield dispersions of flakes of 2D materials was validated by means of an analysis of dispersed flakes for the case-study examples of WS_2_, MoS_2_, and graphene.

**Figure 2:**
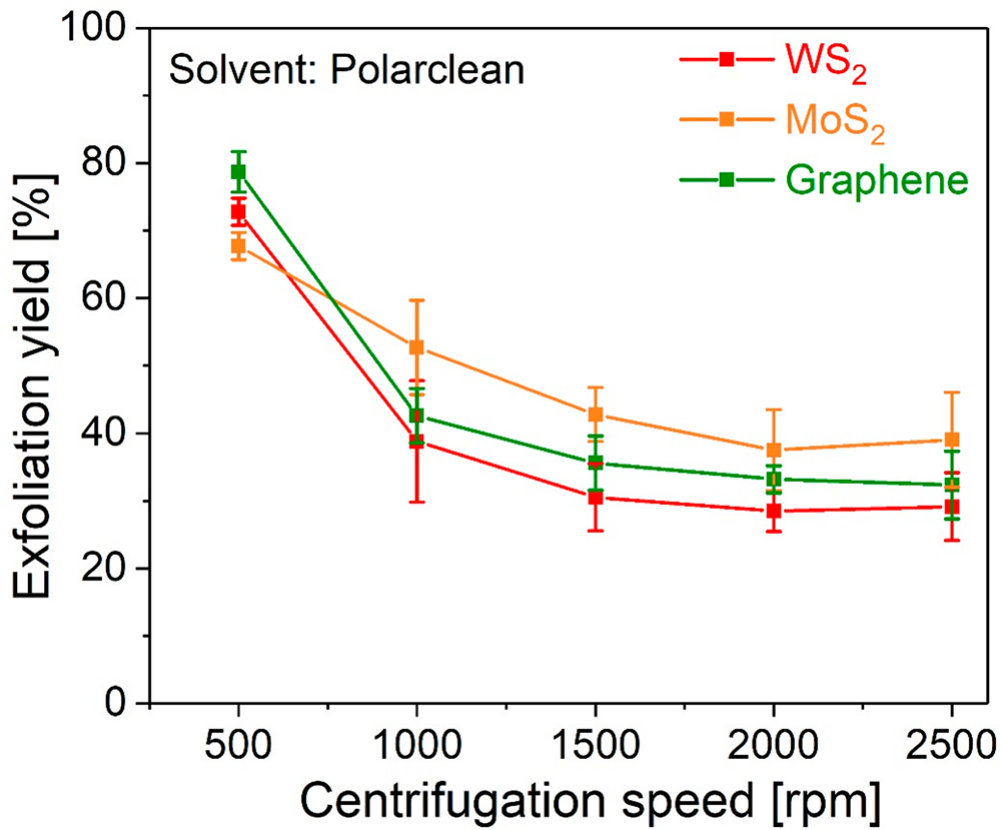
Yield of the Polarclean-assisted LPE as a function of centrifugation speed for WS_2_, MoS_2_, and graphene.

Figure 2 reports the yield of the process as a function of the centrifugation speed, in terms of the amount of flakes in the final dispersion as compared to the initial concentration. For the optimized process, in the case of WS_2_ and graphene, the yield is ∼40% of the initial mass in the final dispersion after a 1000 rpm centrifuge, while the value for MoS_2_ is even higher (50%). For the sake of completeness, we report a comparison of the yields obtained with different solvents in the same operating conditions in the Supporting Information, Figure S2.

**Figure 3:**
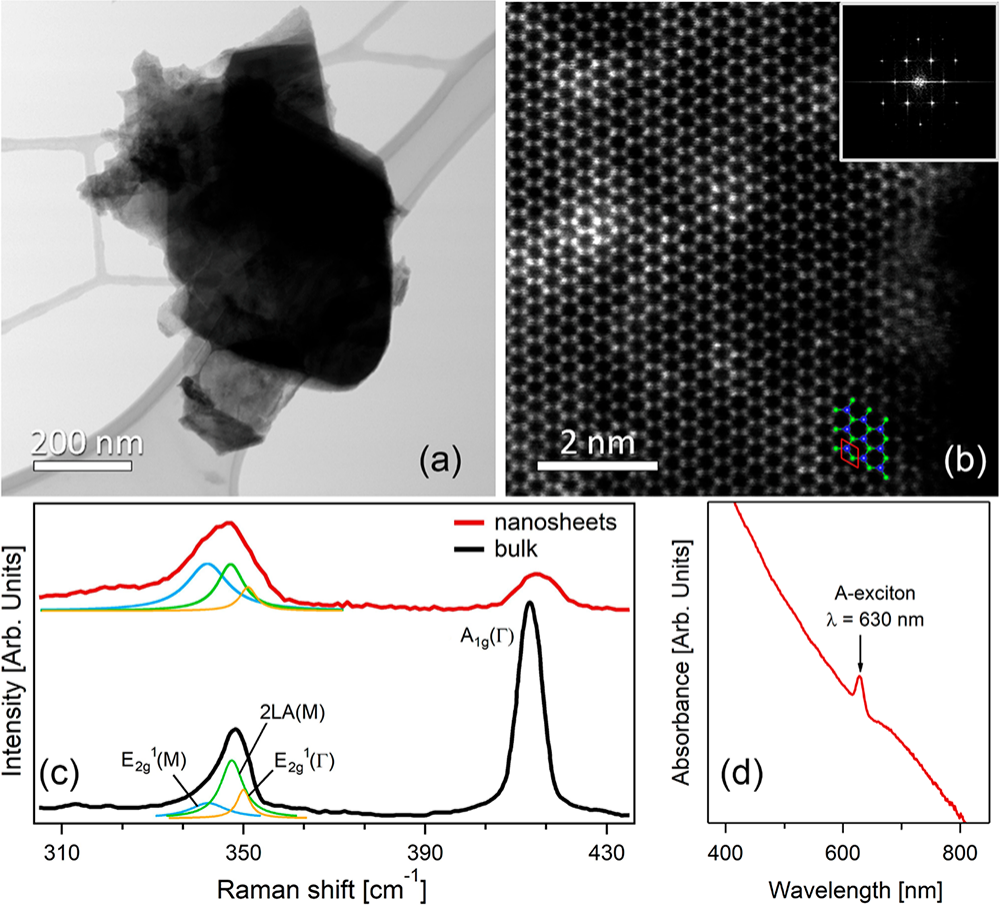
(a) BF-STEM micrograph with different overlapped flakes of WS_2_ transferred on a lacey carbon grid. (b) Atomic-resolution HAADF-STEM micrograph on the side of the same sample in panel a, in correspondence of an isolated flake. A ball-and-stick representation of the WS_2_ atomic structure is overlapped to the experimental micrograph, with W and S atoms depicted in blue and green, respectively, while the unit cell is indicated by red lines. The contrast in intensity of W and S sites is due to their different atomic number.^107^ The inset reports the fast Fourier transform (FFT) of the micrograph. (c) Raman spectra for bulk WS_2_ and for nanosheets exfoliated in the liquid phase using Polarclean solvent. (d) Absorbance spectrum in the 400–800 nm range, showing the A exciton.

To confirm that the exfoliation process only breaks the van der Waals interlayer bonds without deteriorating covalent bonding within the WS_2_ flake, i.e., its crystal structure, we analyzed (i) the atomic structure and (ii) phonon modes by means of scanning transmission electron microscopy (STEM) and Raman spectroscopy. Definitely, atomic-resolution HAADF (high angle annular dark field)-STEM images (Figure 3b) of an exfoliated WS_2_ flake identified in BF (bright field)-STEM (Figure 3a) directly demonstrate that LPE in Polarclean did not induce formation of defects. We also note the absence of defective areas on terraces. The analysis of Raman spectra is fully consistent with STEM analysis. As shown in Figure 3c, the Raman spectrum of WS_2_ is dominated by three major modes: (i) E_2g_^1^(Γ) and (ii) A_1g_(Γ), which are first-order modes, in-plane and out-of-plane, respectively, and (iii) 2LA(M), a second-order longitudinal acoustic mode.^108−110^ Despite the fact that 2LA(M) overlaps E_2g_^1^ (Γ), the fit procedure (Figure 3c) allows the identification of their related components. Indeed, the analysis of the 2LA(M) and A_1g_(Γ) peak intensity ratio, *I*(2LA(M))/*I*(A_1g_(Γ)), is widely recognized as a reliable spectroscopic tool to evaluate the thickness of WS_2_ samples.^111^ In our case, the spectral analysis indicates *I*(2LA(M))/*I*(A_1g_(Γ)) values of ∼0.28 in WS_2_ powder and ∼1.4 in Polarclean-exfoliated WS_2_ flakes. These values correspond to those measured for *I*(2LA(M))/*I*(A_1g_(Γ)) corresponding to bulk WS_2_ (<0.5) and few-layer WS_2_ flakes (>0.5), respectively.^111^

Furthermore, the WS_2_ dispersions in Polarclean were characterized by UV–vis spectroscopy (Figure 3d). The observation of the characteristic absorption at around 630 nm related to the A-exciton, corresponding to the excitonic absorption band originating from gap transition at the K-point of the Brillouin zone,^112^ ensures that the LPE in Polarclean solvent did not modify the electronic structure of WS_2_.

The WS_2_ dispersion had a concentration of ∼0.2 mg/mL (with a yield of ∼40% of the initial mass in the final dispersion) with an estimated value of the optical absorption coefficient α of 1549 ± 50 L/(g m), congruently with previous reports.^52,113,114^ Results for MoS_2_ and graphene are reported in the Supporting Information (Figure S4b,c).

The stability of the dispersions was assessed by taking photographs along 1 week (Supporting Information, Figure S4d–f), finding in all cases that around 80% of the flakes remain in the dispersions even after 1 week.

**Figure 4:**
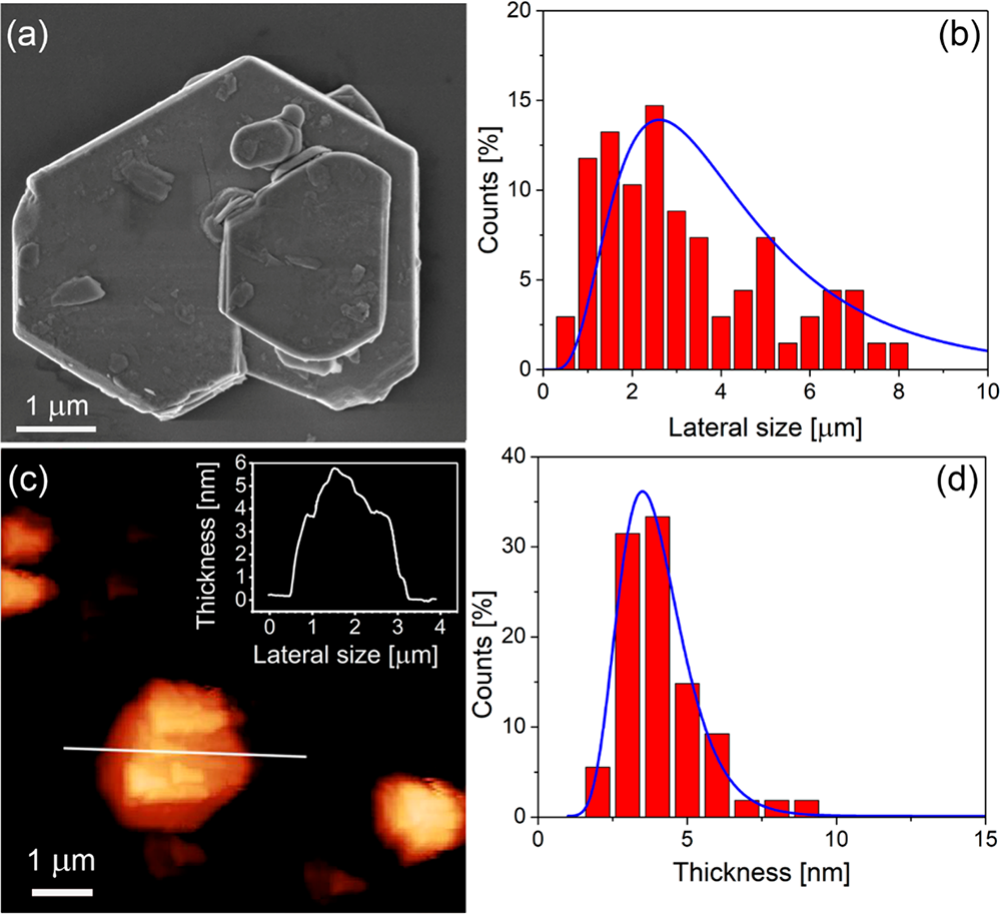
(a) Representative high-resolution SEM image of typical WS_2_ flakes. (b) Analysis of lateral size distribution of WS_2_ flakes determined from SEM images. (c) Representative AFM image of WS_2_ flakes. The height profile along the white solid line is reported in the inset. (d) Analysis of thickness distribution determined from AFM measurements.

Figure 4 reports a statistical analysis of lateral size and thickness of WS_2_ flakes based on images acquired with scanning electron microscopy (SEM) and atomic force microscopy (AFM), respectively. The lateral size and thickness of the WS_2_ flakes approximately follow a log-normal distribution peaked at ∼3 μm (Figure 4c) and ∼4 nm (Figure 4d), respectively. These results allow concluding that Polarclean-assisted LPE provides flakes with an aspect ratio of ∼10^3^.

The performances of Polarclean as an exfoliation medium for 2D materials was directly compared with the case of the most diffuse state-of-the-art solvent, i.e., NMP. Therefore, we performed LPE under the same operating conditions also for NMP (see the Experimental Section for experimental procedures and Figures S5–S7 in the Supporting Information for morphological and physicochemical characterization). While the lateral size is comparable (Figure S5 of the Supporting Information), the statistical analysis on thickness reveals a bimodal distribution for NMP-exfoliated flakes (Figure S5 of the Supporting Information), peaked around 4 and 30 nm, corresponding to thin and thick flakes, respectively. Remarkably, ∼85% of flakes exfoliated by Polarclean have a thickness <5 nm. Considering recent discoveries on the apparent height of monolayer flakes of exfoliated layered materials in AFM experiments with respect to the supporting substrate,^115^ we can infer the predominance of few-layer flakes (1–3 layers) in Polarclean-assisted LPE. Conversely, by using NMP in the same experimental conditions, ∼76% of flakes have thickness >5 nm, thus evidencing a largely incomplete exfoliation of the bulk crystal in NMP-assisted LPE. Congruently, HAADF images of NMP-exfoliated WS_2_ flakes (Figure S7 of the Supporting Information) are consistent with an incomplete exfoliation of the parental bulk crystal, as evidenced by (i) the higher *Z*-contrast and (ii) the multilayered structure imaged in the atomic-resolution HAADF-STEM micrograph in Figure S7f.

**Figure 5:**
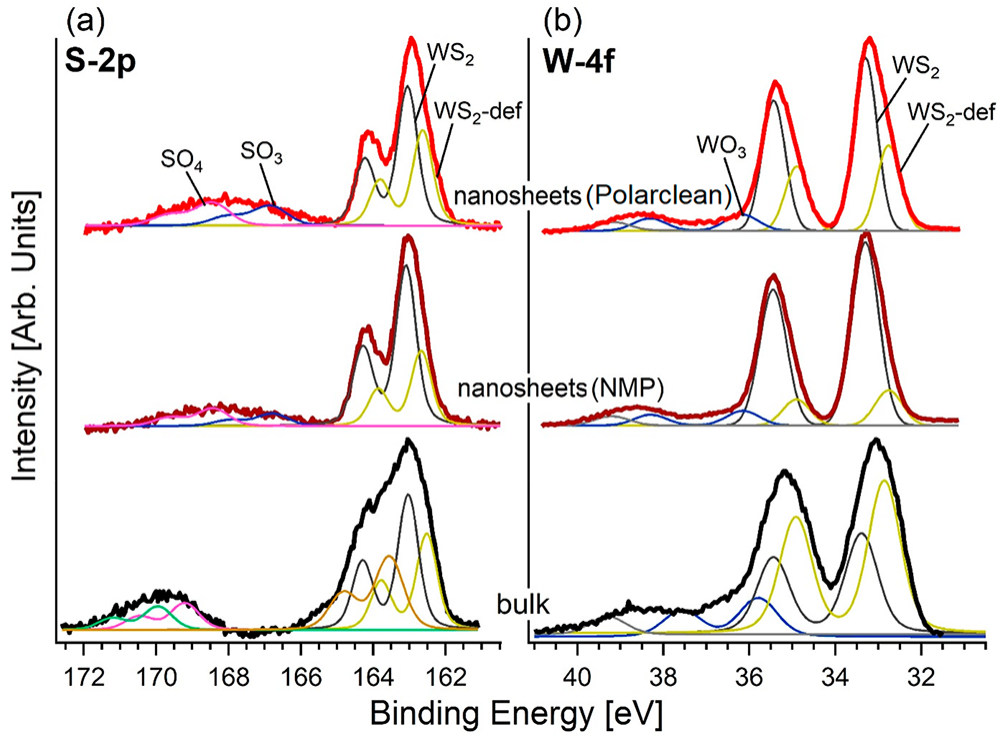
(a) S 2p and (b) W 4f core-level spectra of powder and Polarclean-exfoliated and NMP-exfoliated WS_2_ samples.

To assess eventual modifications in the physicochemical properties of exfoliated flakes, we performed XPS measurements in the region of W 2f and S 2p core levels (Figure 5) for (i) the starting bulk and (ii) exfoliated WS_2_ nanosheets obtained by LPE with both Polarclean and NMP. The W 4f core levels are split in *J* = 5/2 and 7/2 components shifted by 2.1 eV. Specifically, measurements indicate that W 4f core levels have three different contributions arising from WO_3_, WS_2_, and defective WS_2_ (sulfur vacancies) with a binding energy (BE) of 36.1, 33.2, and 32.7 eV for the *J* = 7/2 component, respectively, in agreement with previous works on WS_2_-based systems.^116−118^

Correspondingly, the S 2p core levels are split in *J* = 1/2 and 3/2 components shifted by 1.2 eV. Four well-distinct contributions associated with SO_4_, SO_3_, stoichiometric WS_2_, and defective WS_2_ are observed at BEs of 168.4, 166.7, 163.0, and 162.6 eV for the *J* = 3/2 component, respectively, as in previous reports.^116−118^

Notably, the XPS analysis indicates that the exfoliation process does not induce further oxidation, regardless the increase of the surface/volume ratio when going from bulk WS_2_ to nanosheets. Components related to WO_3_ have only 7.8% and 7.3% of the total areas of the W 4f in Polarclean- and NMP-exfoliated WS_2_, respectively. Similarly, spectral components in S 2p related to SO_4_ and SO_3_ have only ∼8% and 6–9% of the total area for both solvents. Essentially, the physicochemical properties of WS_2_ nanosheets produced by using NMP and Polarclean solvents are similar. The lack of additional spectral contributions in core levels demonstrates that Polarclean-assisted LPE did not alter the electronic properties of the layered material, as also confirmed by XPS measurements for the parental compound MoS_2_ (Supporting Information, Figure S10).

**Figure 6:**
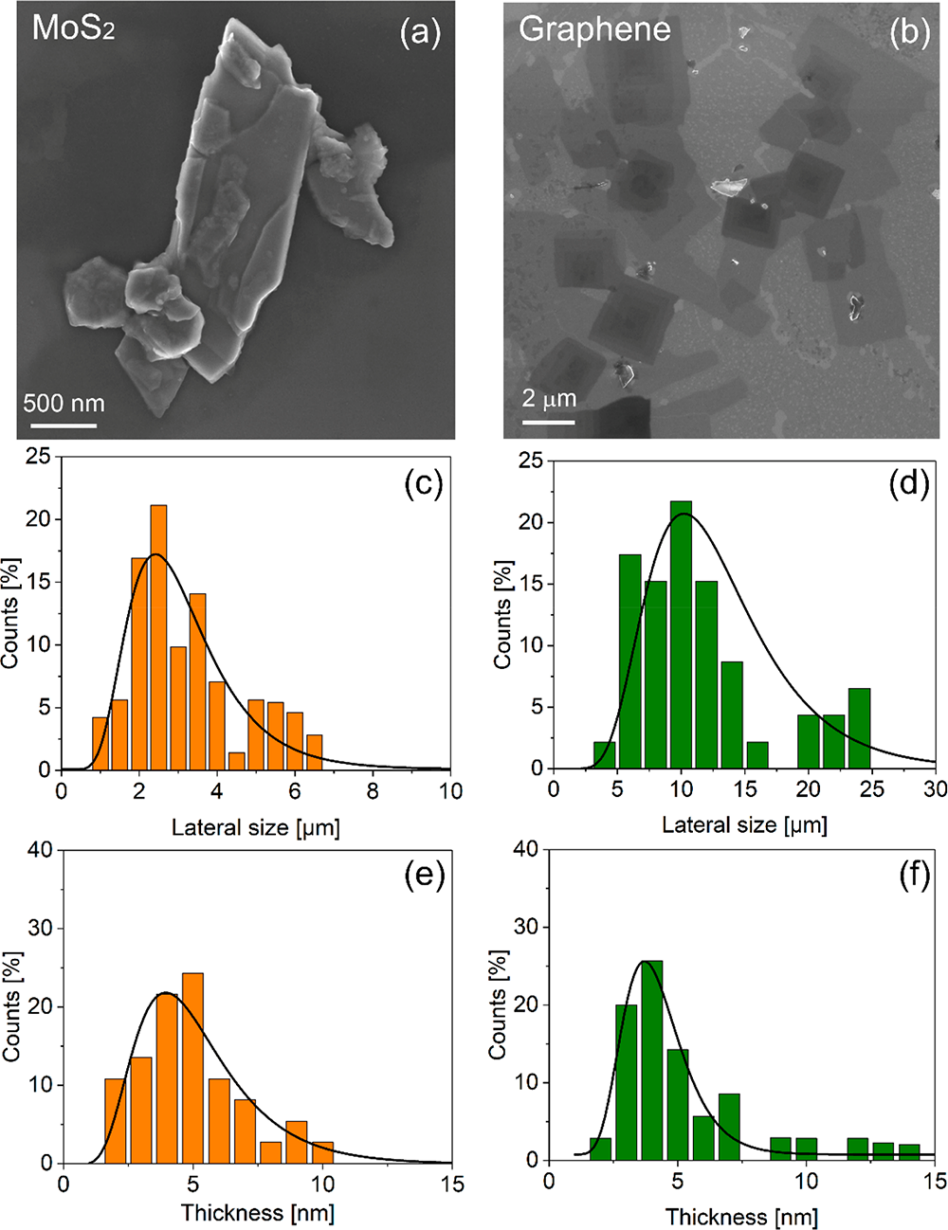
Representative SEM image of isolated exfoliated flakes of (a) MoS_2_ and (b) graphene. Statistical analysis of (c, d) lateral size and (e, f) thickness of (c, e) MoS_2_ and (d, f) graphene flakes, respectively.

In order to validate the extension of the use of Polarclean as the exfoliation medium for layered materials, we demonstrated the efficiency of sonication-assisted LPE for the cases of MoS_2_ and graphene (Figure 6).

Figure 6a,c,e reports a representative SEM image of Polarclean-exfoliated MoS_2_ flakes and the related statistical analysis on lateral size and thickness, respectively. Correspondingly, Figure 6b,d,f are related to graphene flakes exfoliated using Polarclean as the dispersion medium. For the sake of completeness, further morphological and physicochemical characterization of exfoliated MoS_2_ and graphene nanosheets are reported in the Supporting Information, Section S4.

Regarding MoS_2_ exfoliation, statistical analyses on both lateral size and thicknesses (Figure 6c,e) indicate values comparable with the case of WS_2_. Explicitly, the distribution of lateral size is peaked around ∼2.5 μm, while the thickness distribution is centered around 4–5 nm.

**Figure 7:**
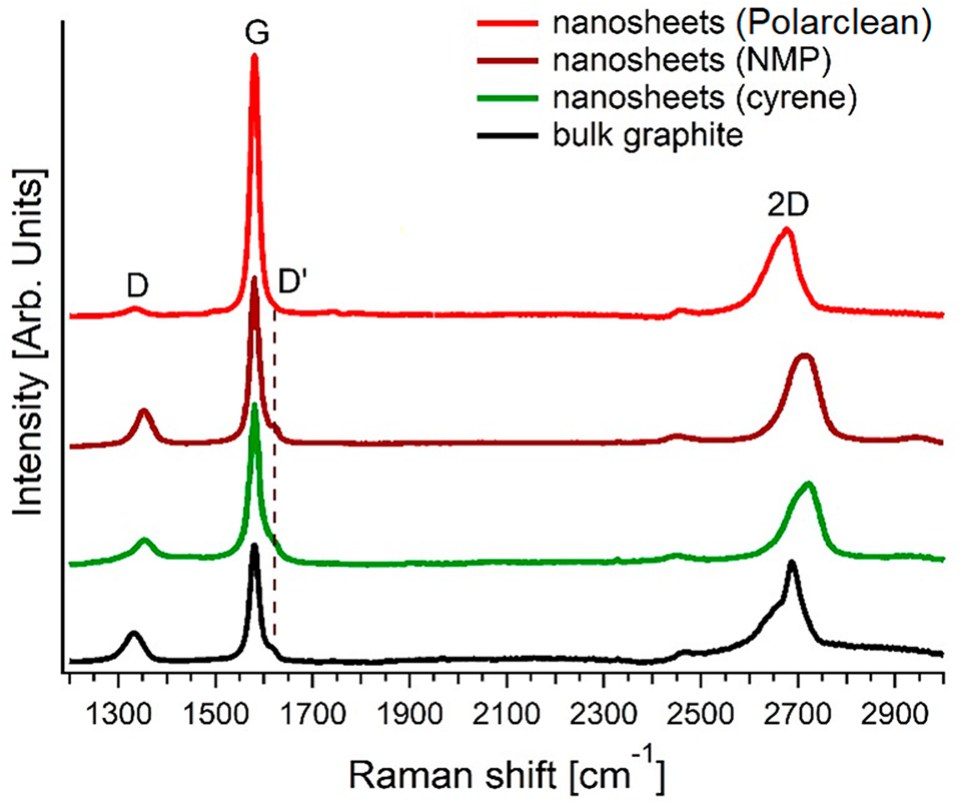
Raman spectrum for graphene exfoliated with Polarclean solvent (brown curve). For the sake of comparison, we report also Raman spectra for NMP-assisted (green curve) and Cyrene-assisted (red curve) LPE exfoliation of graphene (data taken from ref (79)) and, moreover, bulk graphite (black curve). See Figure S12 in the Supporting Information for a comparison extended to other solvents.

Concerning the exfoliation of graphene nanosheets with Polarclean, remarkably, the distribution of lateral size reaches an average value of 10 μm, which is one of the largest reported so far for LPE of graphite.^12,19^ The corresponding Raman spectrum (Figure 7) displays D and G bands at 1331 and 1581 cm^–1^. We recall that, while the G peak arises from the E_2g_ optical phonon of graphene,^119^ the D band is originated by breathing modes of six-atom rings and requires a defect for its activation.^120^ Therefore, the *I*(D)/*I*(G) ratio is a widely recognized probe of structural defects in the graphene sheet^121^ (see also the Supporting Information, Section S5). Notably, the ratio of the intensity of D and G Raman-active bands in few-layer graphene exfoliated through Polarclean is *I*(D)/*I*(G) = 0.07 ± 0.01. Definitely, we estimate a density of defects as low as (8 ± 2) × 10^9^ cm^–2^, which is consistent with the high crystalline order of exfoliated graphene flakes (without evidence of defects) imaged by high-resolution TEM (HR-TEM) in Figure S11 of the Supporting Information. For the sake of comparison, from the *I*(D)/*I*(G) analysis in Raman spectra (Figure 7 and Figure S12 of the Supporting Information), we also estimated the density of defects for graphene exfoliated with NMP,^79,122^ Cyrene,^79^ IPA,^123^ DMF,^124^ acetone/water,^125^ ethanol/water,^126^ TEA,^73^ and aqueous solution of urea,^74^ finding values of (6 ± 2) × 10^10^, (5 ± 2) × 10^10^, (1.0 ± 0.3) × 10^11^, (9 ± 3) × 10^10^, (4 ± 1) × 10^10^, (2.6 ± 0.7) × 10^11^, (6 ± 2) × 10^10^, and (7 ± 2) × 10^10^ defects/cm^2^, respectively. Evidently, graphene flakes exfoliated with Polarclean exhibit a density of defects inferior by approximately 1 order of magnitude with respect to LPE assisted by other solvents.

The analysis of the intensity of the D′ band can provide further indication on the density of defects. Similarly to the D band, the D′ mode is a double resonance originated by the transverse optical (TO) phonons around the K or K′ points in the first Brillouin zone, and it is activated by defects, although it involves an intravalley rather than intervalley process.^121^ Remarkably, the intensity of the D′ band at 1615 cm^–1^ is suppressed for the case of Polarclean-assisted LPE of graphene, in contrast with the case of other solution processing methods (Figure 7 and Figure S12 of the Supporting Information).

## Conclusions

We have proven that Polarclean is an efficient green solvent for the production of layered materials by LPE. In particular, sonication-assisted LPE provided distributions of lateral size of 4, 3, and 10 μm and thickness of 4, 4, and 5 nm for WS_2_, MoS_2_, and graphene. Concurrently, the amount of few-layers sheets (below 5 nm) in dispersions in Polarclean is higher by ∼350% compared to LPE with NMP. Correspondingly, the density of defects of graphene flakes produced by LPE is reduced by 1 order of magnitude by using Polarclean, as evidenced by the *I*(D)/*I*(G) ratio in Raman spectra of graphene as low as 0.07 ± 0.01. The superior performances in LPE, together with the absence of any toxicity issue and its biodegradability, make Polarclean an ideal candidate for sustainable large-scale production of 2D materials. Naturally, Polarclean can also replace solvents commonly employed for other processing methods beyond sonication, such as shear mixing^127^ or wet-jet mill,^128^ particularly promising for industrial scale-up. The efficiency of the Polarclean-based LPE process is crucial in order to combine intrinsic benefits for environmental health and safety with optimization of performances. Undeniably, the introduction of a green solvent for LPE also will expand the growing market of 2D materials toward fields to date nearly unexplored (e.g., recovery of minerals from seawater, concentration of fruit juices, production of drinking water, etc.), as a result of the toxicity of state-of-the-art solvents for LPE, with subsequent superb impact on the commercial potential of their technological applications.

## Experimental Section

### Materials

WS_2_ (CAS number 12138-09-9), MoS_2_ (CAS number 1317-33-5), and graphite (CAS number 7782-42-5) were purchased from Sigma-Aldrich and used without further purification. Related particle size distributions are reported in the Supporting Information, Figure S1. Absolute ethanol, *N*-methyl-2-pyrrolidone (NMP), triethanolamine (TEA), and urea were purchased from commercial chemical suppliers. Methyl-5-(dimethylamino)-2-methyl-5-oxopentanoate (Rhodiasolv Polarclean) was provided by Rhodiasolv, Solvay Novecare, Paris.

### Exfoliation of Layered Materials

A 0.05 g portion of a powder of WS_2_, MoS_2_, and graphite was dispersed in 40 mL of Rhodiasolv Polarclean and sonicated for 3 h in a bath sonicator (Elmasonic P working at 37 kHz) in a thermostat bath to prevent excessive temperature rise (*T* ≤ 25 °C). Beside exfoliation, in order to physically remove Polarclean, several centrifugations were carried out. After a first centrifugation at 5000 rpm, supernatant was discarded and substituted with an analogous amount of ethanol. After this step, 3 successive centrifugations were performed to remove solvent residuals, with a last centrifugation at 1000 rpm aimed at separating thinner flakes from thick and unexfoliated material. Finally, the supernatant was collected for characterization.

### Characterization

STEM investigation was performed with a JEOL ARM200F Cs-corrected microscope, equipped with a cold-field emission gun with an energy spread of 0.3 eV and operating at 60 keV. The probe size was 1.1 Å at 60 kV. Micrographs were acquired in BF and in Z-contrast mode by HAADF.

Field emission scanning electron microscope (FESEM) experiments were carried out at the Microscopy Centre of University of L’Aquila with a Gemini SEM 500 instrument, at an accelerating voltage of 2 kV. AFM measurements were performed in air tapping mode with a Veeco Digital D5000 system, using tips with a spring constant of 3 N/m and resonance frequencies between 51 and 94 kHz.

Raman spectra were acquired using a micro-Raman spectrometer (μRS) (LABRAM spectrometer, λ = 633 nm, Horiba-Jobin Yvon, Kyoto, Japan) equipped with a confocal optical microscope (100× MPLAN objective with 0.9 numerical aperture and 0.15 mm work distance). The spatial resolution was ∼1 μm, while the energy resolution was ∼2 cm^–1^.

Optical absorption spectra of WS_2_, MoS_2_, and graphene dispersions achieved after Polarclean-assisted LPE were measured by using a UV–vis spectrometer (PerkinElmer, Lambda 750) with a 1 cm quartz cuvette. Moreover, UV–vis spectra of differently diluted dispersions were used to estimate optical absorption coefficients by applying Lambert–Beer’s law. To estimate the concentration, dispersions were filtered. By measuring the filtered mass, we evaluated the concentration of flakes after exfoliation and centrifugation.

XPS measurements were performed using a PHI 1257 spectrometer, equipped with a monochromatic Al Kα source (*h*ν = 1486.6 eV) with an experimental resolution of 0.25 eV.

## Supporting Information Available

Theoretical model on LPE, characterization of the dispersions, comparison between Polarclean- and NMP-assisted liquid-phase exfoliation, and validation of Polarclean for liquid-phase exfoliation of MoS_2_ and graphene (PDF)
