# Supplementary material for: Sustainable Liquid-Phase Exfoliation of Layered Materials with Nontoxic Polarclean Solvent
Source: ACS Sustain Chem Eng. 2020 Dec 14;8(51):18830–40. doi: 10.1021/acssuschemeng.0c04191 (PMC8018326; doi:10.1021/acssuschemeng.0c04191)
Supplement: Supplementary file 1 — sc0c04191_si_001.pdf [file sc0c04191_si_001.pdf]

Supporting Information for the manuscript

Sustainable liquid-phase exfoliation of layered materials with non-toxic Polarclean solvent

Valentina Paolucci[1,*], Gianluca D'Olimpio[2], Luca Lozzi[2], Antonio M. Mio[3], Luca Ottaviano[2], Michele Nardone[2], Giuseppe Nicotra[3], Patrice Le-Cornec[4,*], Carlo Cantalini[1, *], Antonio Politano[2,3, *]

1 Department of Industrial and Information Engineering and Economics, Via G. Gronchi 18, University of L'Aquila, I-67100 L'Aquila, Italy

2 Department of Physical and Chemical Sciences, University of L'Aquila, via Vetoio, 67100 L'Aquila (AQ), Italy

3 CNR-IMM Istituto per la Microelettronica e Microsistemi, VIII strada 5, I-95121 Catania, Italy

4 Solvay Novecare, 93308 Auberviliers, France

Supporting Information content:

Number of pages: 21 (S1 to S21)

Number of figures: 5 (Figure S1 to Figure S12)

Number of tables: 2 (Table S1 to Table S2)

S1. Theoretical models on liquid-phase exfoliation

Considering that, according to Table I in the main text, the surface tension of Polarclean at T=298 K is 38 mN m^{-1} and that of NMP is 40 mN m^{-1}, data in Table S1 are displayed to assess the compatibility of these solvents with the most studied layered materials. Apparently, the reported values of surface energy are higher than surface tension of the solvent, but it is worth noting that surface energies of liquids are in general 30 mN m^{-1} higher than the corresponding surface tensions at that temperature. As a matter of fact, it was demonstrated [1] that this difference of 30 mN m^{-1} arises essentially from the definition of surface tension (σ_S):

$$\sigma_S = (E_{Sur} - TS_{Sur}) \qquad (S1)$$

where E_{Sur} corresponds to surface energy and S_{Sur} surface entropy. Given that S_{Sur} for liquids generally is around 0.1 mN m^{-1} and considering that all these values are taken at 298 K, the difference of about 30 mN m^{-1} is confirmed.

Table S1. Comparison of Surface Energies and Hansen Solubility Parameters of the most studied layered materials.

	Surface Energy	Hansen Solubility Parameters		
	E_{sur} [mN m^{-1}]	δ_d [$MPa^{1/2}$]	δ_p [$MPa^{1/2}$]	δ_H [$MPa^{1/2}$]
WS_2 [2]	≈ 75	16-18	5-14	2-19
MoS_2 [2]	≈ 70	17-19	6-12	4.5-8.5
Graphite [3]	≈ 62	≈18	≈9.3	≈7.7
Boron Nitride[2]	≈ 65	17-19	4-10	4-10

However, a more accurate modelling exploring the relationships between surface tension and dispersibility of the layered materials, still represents an open question.

The first works on liquid-phase exfoliation tried to relate the yield with surface tension of the adopted solvent. Yield is maximized by the minimization of the enthalpy of mixing per unit volume ΔH/V, which, in turn, is connected to the Helmholtz free energy of solvent (F_{solv}) and of the Helmholtz free energy layered materials ($F_{layered}$), the thickness of the flakes ($T_{layered}$), and the volume fraction (φ) following [2, 4]:

$$\frac{\Delta H}{V} \sim \frac{2}{T_{layered}} \left(\sqrt{F_{solv}} - \sqrt{F_{layered}}\right)^2 \phi \qquad \text{(S2)}$$

With

$$F_{layered} = (\sigma_s - TS_{Sur}) \qquad \text{(S3)}$$

where σ_s is the surface energy and S_{Sur} surface entropy.

The great limitation of this approach is that it considers as null the interface energy between the solvent and the materials to be exfoliated. Therefore, it works only under very limited regimes or in some specific cases, but it is far from being a general rule.

A successive work [5] suggests that higher yield can be achieved by combining solvents and materials, so that

$$\sigma_s - \sigma_l - \sigma_{sl} > 0 \quad \text{(S4)}$$

Accordingly, the yield is maximized whenever $\sigma_s > \sigma_l + \sigma_{sl}$

However, a more complete theoretical model is developed in Refs. [6-7], based on the minimization of the interfacial surface tension between the solid and the liquid. Within the framework of this model, the surface tension has two components: (i) polar and (ii) dispersive due to long-range interactions. By selecting the most opportune ratios between these quantities in the solvent and in the layered

materials, the yield is maximized based on the minimization of the surface tension. Specifically, according to ref. [6]:

$$\sigma_{sl} = \sigma_s + \sigma_l - 2\left(\sqrt{\sigma_s^d \cdot \sigma_l^d} + \sqrt{\sigma_s^p \cdot \sigma_l^p}\right) = \sigma_s^d + \sigma_s^p + \sigma_l^d + \sigma_l^p - 2\left(\sqrt{\sigma_s^d \cdot \sigma_l^d} + \sqrt{\sigma_s^p \cdot \sigma_l^p}\right)$$

(S5)

where σ_s is the surface tension of the solid (i.e., materials to be exfoliated) and σ_l is the surface tension of the liquid (i.e., the solvent). The superscripts “p” and “d” stand for polar and dispersive components, respectively. σ_{sl} is the interfacial surface tension between the material and the solvent, which should be minimized to improve the efficiency of the exfoliation.

Equation (S5) can be also expressed as:

$$\sigma_{sl} = \left(\frac{\sigma_s^p}{\sigma_s^d} + 1\right)\sigma_s^d + \left(\frac{\sigma_l^p}{\sigma_l^d} + 1\right)\sigma_l^d - 2\left(\sqrt{\frac{\sigma_l^p \sigma_s^p}{\sigma_l^d \sigma_s^d}} + 1\right)\sqrt{\sigma_l^d \sigma_s^d} =$$

$$= \left(\sqrt{\sigma_s^d}\sqrt{\frac{\sigma_s^p}{\sigma_s^d} + 1} - \sqrt{\sigma_l^d}\sqrt{\frac{\sigma_l^p}{\sigma_l^d} + 1}\right)^2 + 2\sqrt{\sigma_l^d \sigma_s^d}\left(\sqrt{\frac{\sigma_l^p}{\sigma_l^d} + 1}\ \sqrt{\frac{\sigma_s^p}{\sigma_s^d} + 1} - \sqrt{\frac{\sigma_l^p \sigma_s^p}{\sigma_l^d \sigma_s^d}} - 1\right) \quad \text{(S6)}$$

By inspecting Eq. (S6), it is evident that in order to minimize interfacial surface tension of 2D materials and solvents, the following conditions should be verified (i) $\sigma_s^d \sim \sigma_l^d$ and (ii) $\sigma_s^p \sim \sigma_l^p$ and, consequently, $\frac{\sigma_s^p}{\sigma_s^d} \sim \frac{\sigma_l^p}{\sigma_l^d}$

In conclusion, the scientific community has not reached consensus yet on a specific theoretical model relating yield and surface tension, although that one in Refs. [6-7] appears by far the most accurate.

S2. Characterization of the WS_2, MoS_2 and graphene dispersions

Few layers dispersions of WS_2, MoS_2 and graphene were obtained by Liquid Phase Exfoliation of commercial powders. Particle size distribution of the starting materials is reported in Fig. S1.

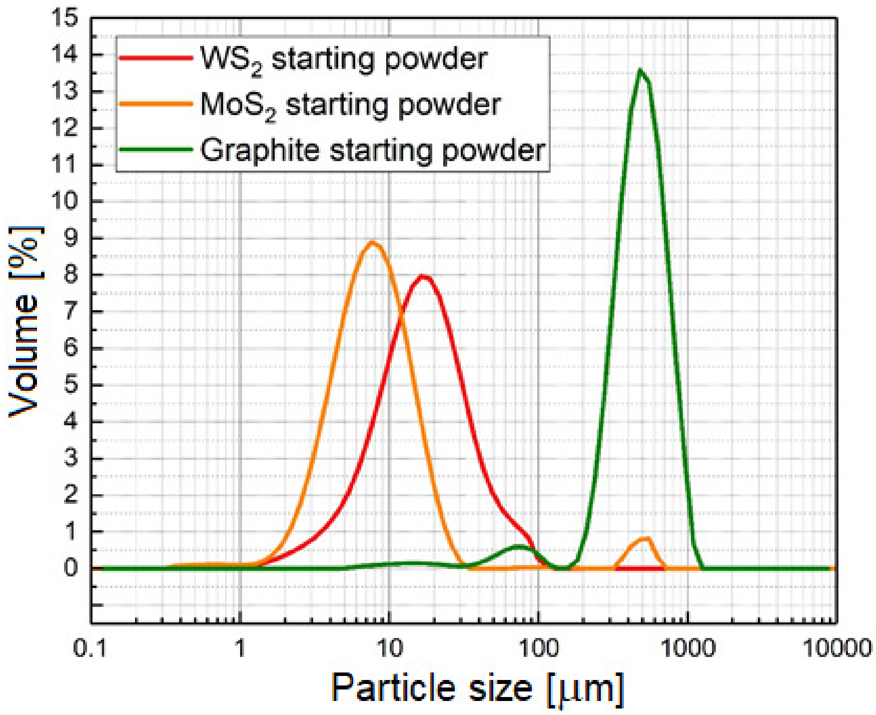

Figure S1. Particle size distribution of the starting commercial powders of WS_2, MoS_2 and graphene.

To provide a straightforward comparative evaluation regarding the yield of LPE by adopting different processes, the exfoliation of WS_2 powder was performed in the same experimental conditions with Polarclean, NMP and aqueous dispersion of urea. The yield was calculated as as a function of the centrifugation speed, in terms of the amount of flakes in the final dispersion as compared to the initial concentration. The results, reported in Figure S2, indicated superior performances of Polarclean as compared to the state-of-the-art solvent (NMP) and recently proposed *green* alternatives (TEA and Urea), confirming its potential application in LPE.

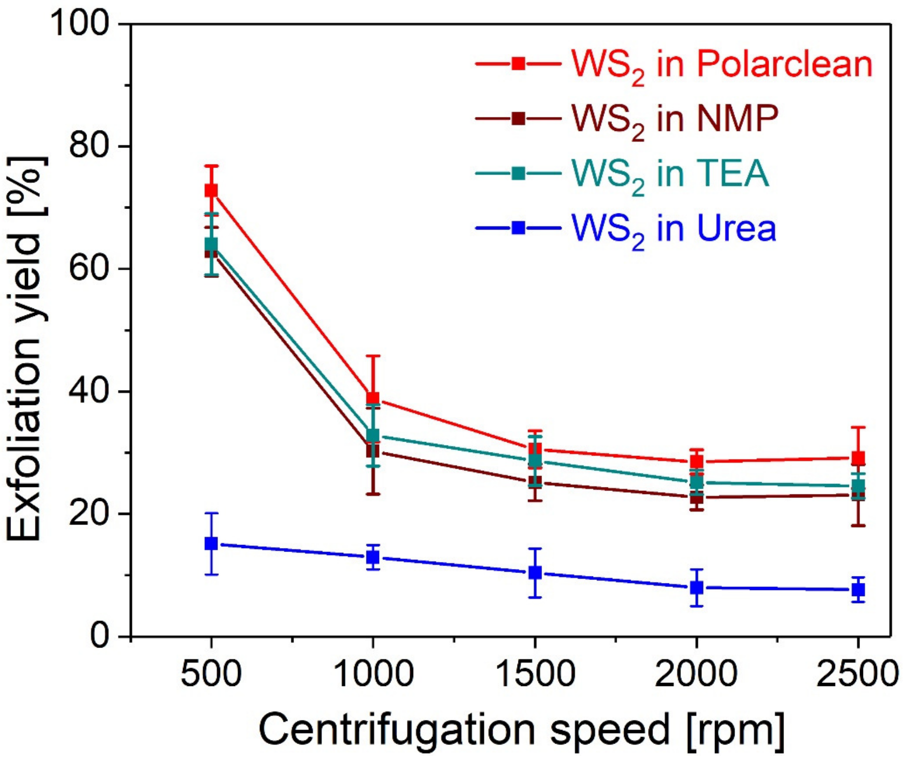

Figure S2. Comparison of the yields of WS_2 exfoliation obtained with different solvents (Polarclean, NMP, TEA and aqueous dispersion of urea) in the same operating conditions.

To investigate the presence of residuals in the dispersion after exfoliation, we measured core levels by XPS. Considering the composition of Polarclean ($C_9H_{17}NO_3$), nitrogen was selected as fingerprint for adsorbed species on the surface of the exfoliated layered materials. Actually, carbon, hydrogen and oxygen are inevitably related to graphene or airborne contamination, adsorbed water from ambient humidity and oxidation.

As shown in Fig. S3, by means of X-ray photoelectron spectroscopy (XPS), we were unable to detect a signal for N-1s. Considering the surface sensitivity of XPS, we can ensure that the surface content of N atoms is extremely reduced. In term of N coverage (θ_N), we can definitely affirm that it is below the sensitivity of the XPS technique under our experimental conditions, namely <0.03 monolayer. In conclusion, we can affirm that only trace residuals of Polarclean are present on the surface of exfoliated nanosheets.

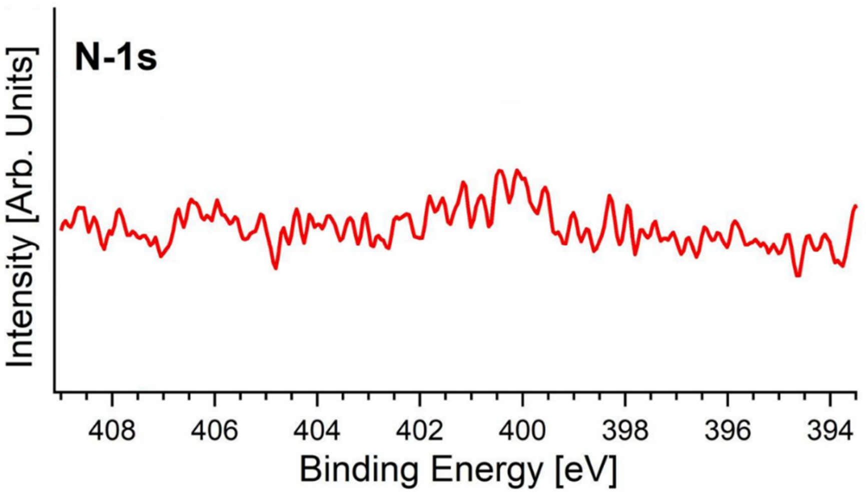

Figure S3. XPS spectra of N 1 s core level of WS_2 flakes showing the absence of any feature related to residual contamination.

The flakes-containing dispersions were then characterized by UV-Vis spectroscopy in order to calculate absorption coefficient and solution concentration. After preparing some differently diluted dispersions, concentration was evaluated by applying the Lambert-Beer's law:

$$A = \alpha C l \quad \text{(S2)}$$

where A represents the absorbance, α the absorption coefficient, l refers to the optical path length and C is the concentration. Figure S4 reports A as a function of solution concentration at a fixed wavelength (λ = 630 nm for WS_2, 670 nm for MoS_2 and 660 nm for graphene, respectively). The fit procedures enable estimating the absorption coefficient α, which corresponds to the slope of the dashed line in the insets of Figs. S4, panels a-c. The calculated values of α are 1549±50 L $g^{-1}m^{-1}$ for WS_2, 1720±20 L $g^{-1}m^{-1}$ for MoS_2 and 2321±20 L $g^{-1}m^{-1}$ for graphene, congruently with previous reports [3, 8-9].

The stability of the dispersions was assessed by taking photographs along one week (Supporting Information, Figure S4 d-f), finding in all cases that around 80% of the flakes remained in the dispersions even after one week.

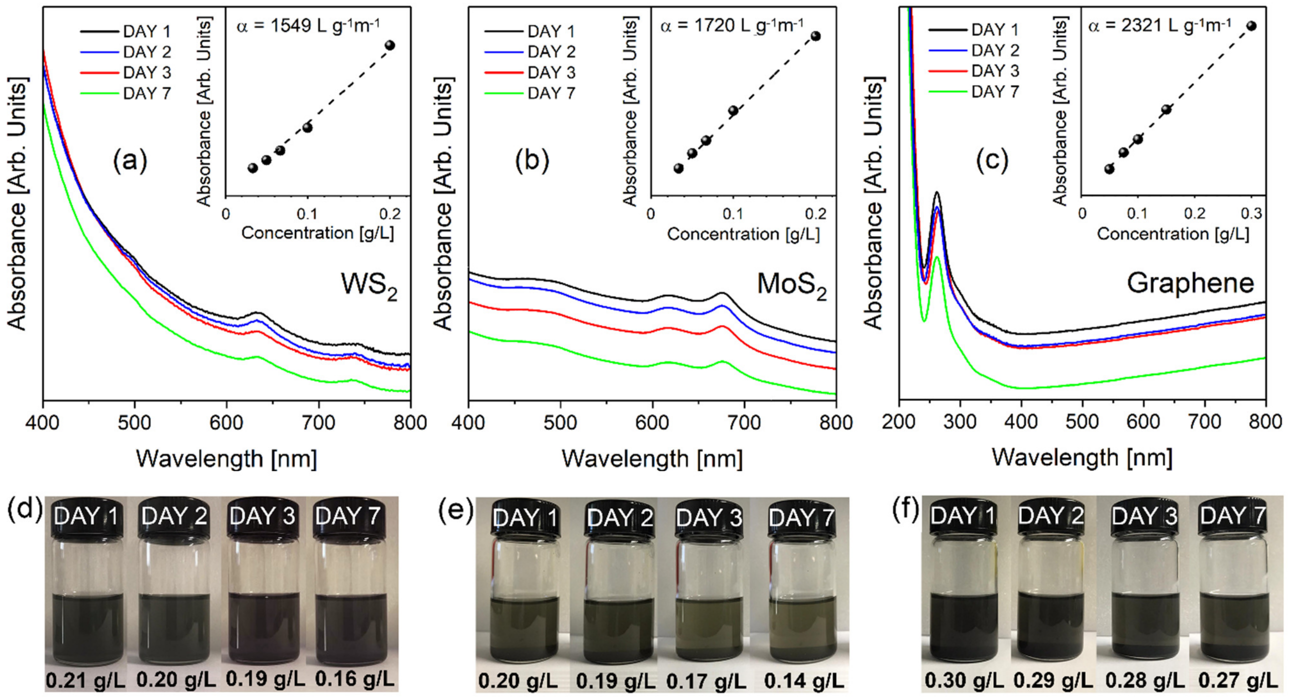

Figure S4. UV-Vis spectra of the Polarclean-exfoliated (a) WS_2, (b) MoS_2 and (c) graphene dispersions collected after 1, 2, 3 and 7 days after exfoliation. Insets report the optical absorbance of day 1 (at 630 nm for WS_2, 670 nm for MoS_2 and 660 nm for graphene) as a function of concentration for estimating the absorption coefficient. Panels (d-f) report photographs of the dispersions of (d) WS_2, (e) MoS_2 and (f) graphene taken along one week to assess their stability.

S3. Comparison with NMP-assisted liquid-phase exfoliation

Considering that NMP represents, so far, the most utilized solvent for LPE, it was used in this work in the same experimental conditions described in Methods section in the main text in order to compare the results achieved for Polarclean.

Figure S5 shows the microstructural characterization of the NMP-exfoliated WS_2 flakes. Specifically, Figs. S5a-b display a representative SEM image and the related statistical analysis. Similarly, typical AFM measurement and statistical thickness distribution are reported in Figs. S5c and S5d, revealing

uncomplete exfoliation of WS_2, thus meaning that longer and possibly more powerful sonication is needed with NMP to achieve the superior outcome given by Polarclean use. STEM measurements of Figs. S6 and S7 confirmed these results. Low-magnification BF-STEM and related atomic-resolution HAADF-STEM in Fig. S6 show WS_2 flakes with high crystallinity and no evidence of defects, except for an amorphous region at flake's edge, as in Fig. 3b for Polarclean-assisted exfoliation. However, the comparison of low- and high-resolution HAADF images of Polarclean-exfoliated and NMP-exfoliated WS_2 flakes in Fig. S7 confirmed the incomplete exfoliation obtained with NMP.

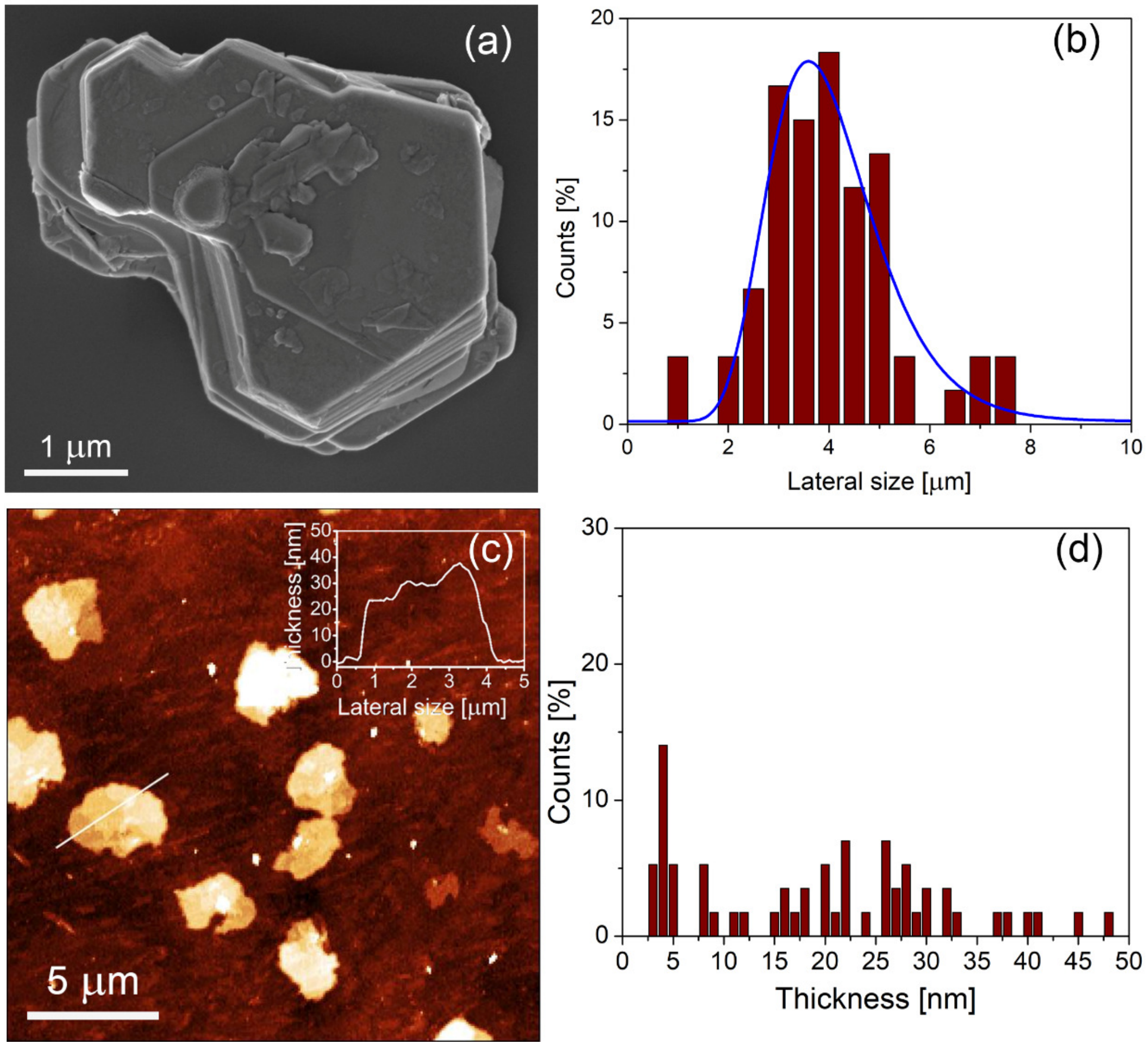

Figure S5. (a) Representative high-resolution SEM image of an isolated WS_2 flake exfoliated in NMP; (b) Distribution of lateral sizes of WS_2 flakes exfoliated with NMP; (c) Representative AFM image of WS_2 flakes. Height profile of along the white solid line is also reported in the inset; (d) Statistical analysis corresponding to thickness distribution of WS_2 flakes exfoliated with NMP solvent, obtained based on AFM measurements.

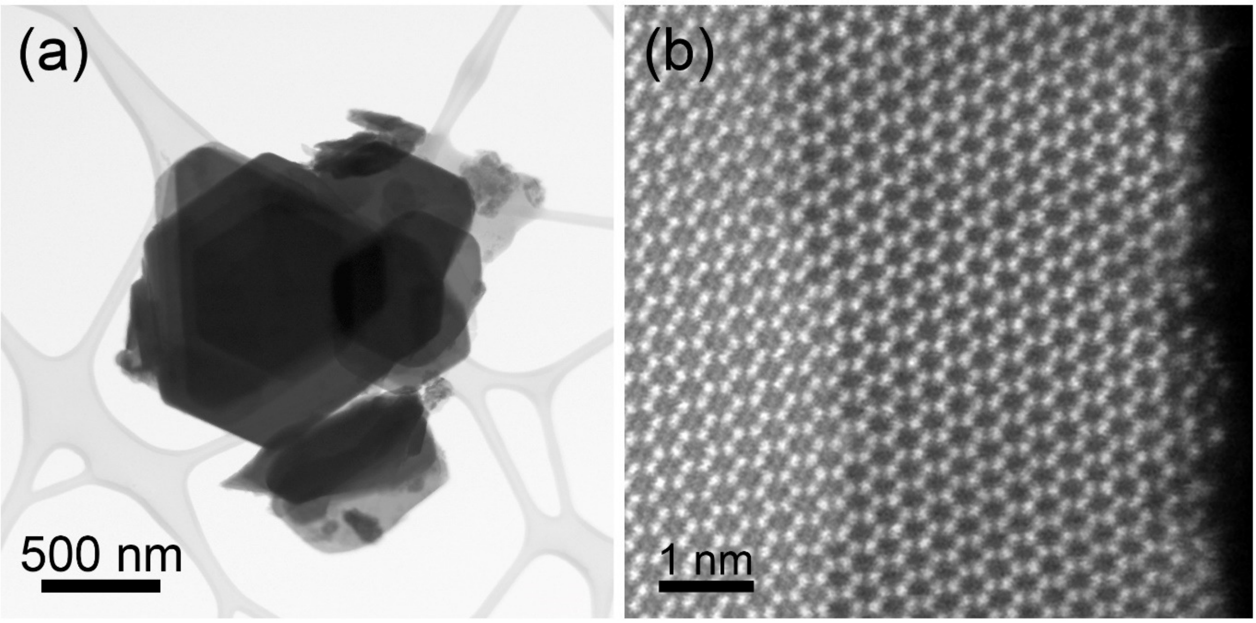

Figure S6. (a) BF-STEM micrograph with different overlapped flakes of WS_2 exfoliated in NMP transferred on a lacey carbon grid. (b) Atomic-resolution HAADF-STEM of one edge of the flake shown in (a).

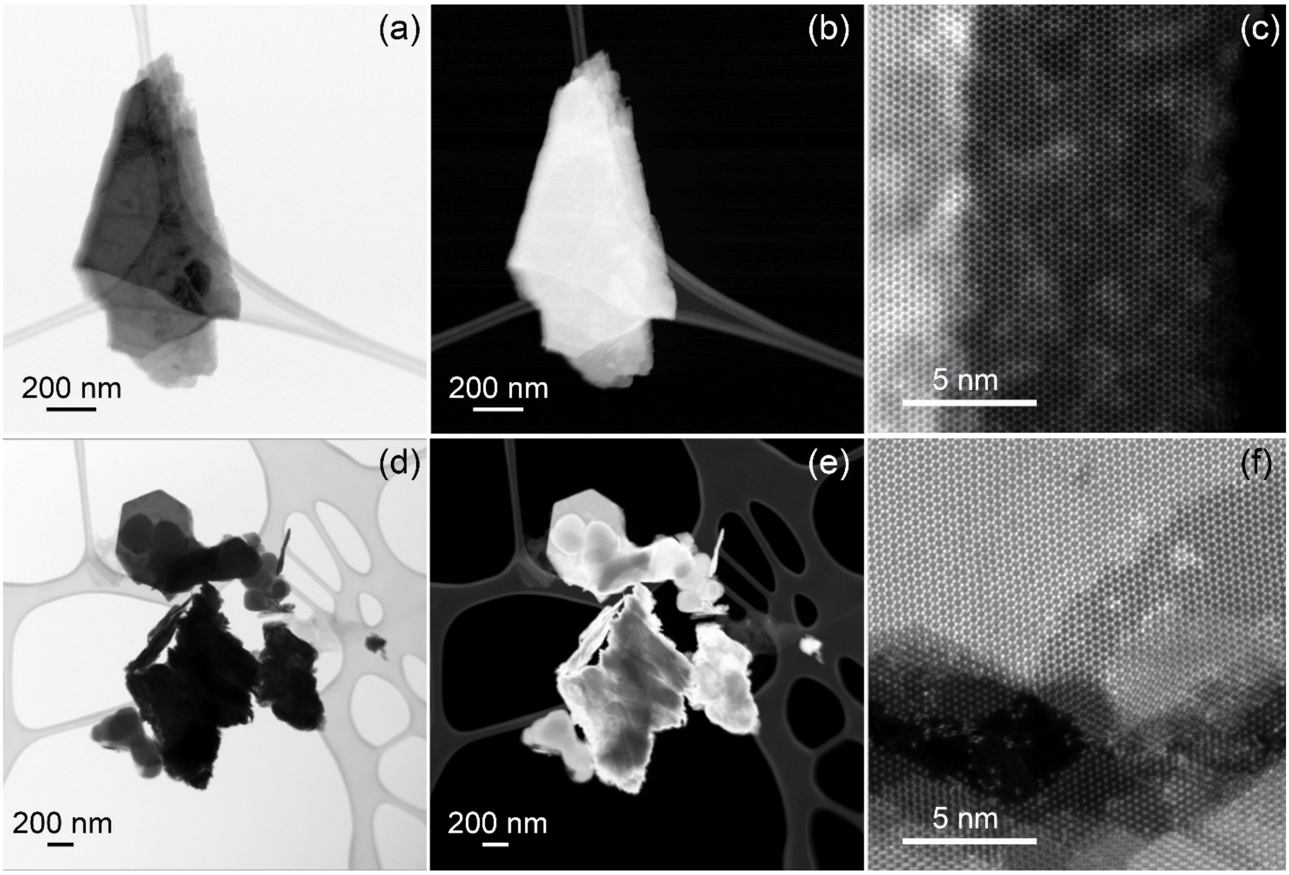

Figure S7. (a) BF-STEM micrograph and corresponding (b) low-resolution HAADF-STEM and (c) atomic-resolution HAADF-STEM micrograph of WS_2 flakes exfoliated in Polarclean; (d) BF-STEM micrograph and corresponding (e) low-resolution HAADF-STEM and (f) atomic-resolution HAADF-STEM micrograph of WS_2 flakes exfoliated in NMP.

S4. Validation of Rhodiasolv©Polarclean for liquid-phase exfoliation of MoS_2 and graphene

The characterizations reported in Section S4 were carried out on MoS_2 and graphene dispersions to extend the use of Polarclean to other materials, in the same solution processing procedure employed for WS_2. Regarding MoS_2, Fig. S8 b, which is a magnification of an edge of the flake shown in Fig. S8a, reveals a three-layered structure and confirms the absence of oxidation on terraces, while a defective region, less than 1 nm large, is observed at the edge of the flake. This evidence is also confirmed in Fig. S9, which is related to the edge of another flake.

Figure S8c reports a representative AFM image of MoS_2 flakes with a typical thickness profile along the white line.

To evaluate the effects of Polarclean-assisted liquid-phase exfoliation on the electronic properties of the MoS_2 nanosheets, we measured absorption spectra by UV-Vis spectroscopy (Fig. S8d). We observe the two characteristic excitonic bands, originated from the interband excitonic transition at the K point of the Brillouin zone [10], located at ~610 (B-exciton) and ~670 (A-exciton) nm [11].

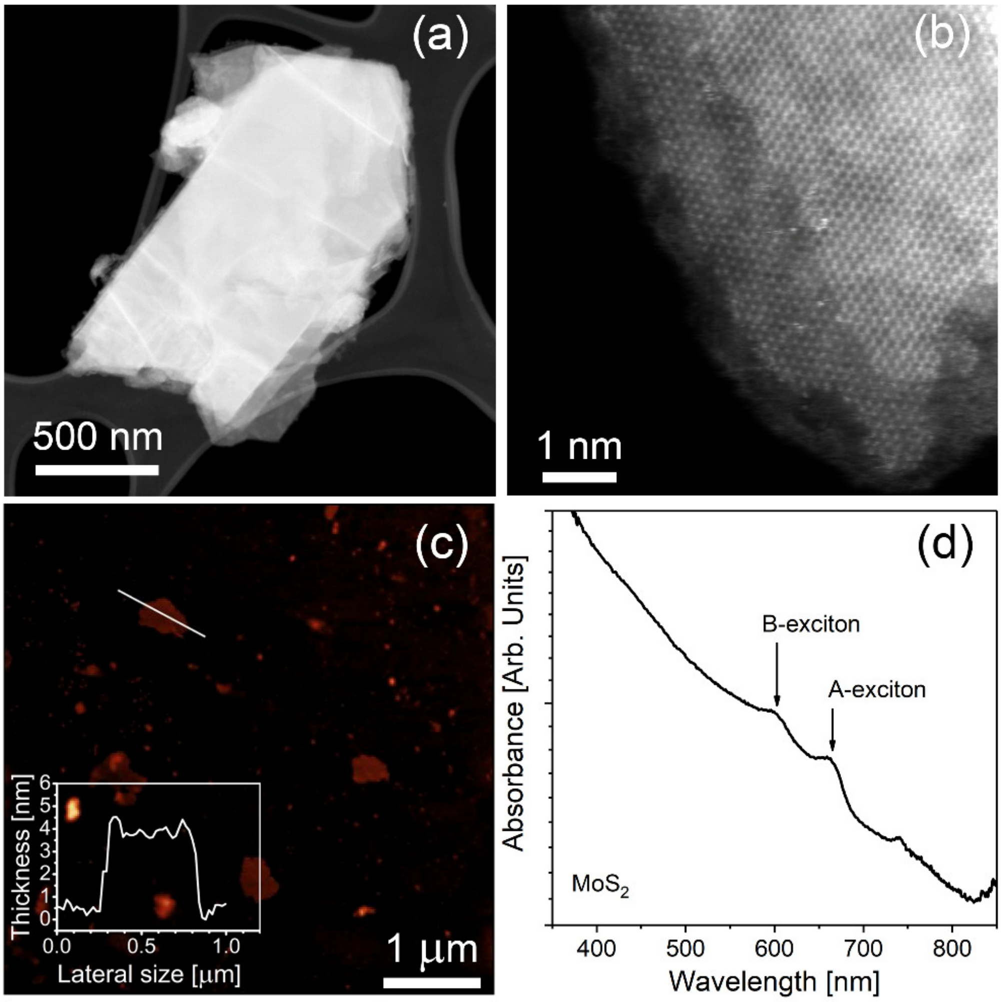

Figure S8. (a) HAADF -STEM image of a MoS_2 flake exfoliated with Polarclean (b) HRTEM of one edge of exfoliated MoS_2 flake. (c) Representative AFM image of a MoS_2 flake exfoliated with Polarclean and (d) UV-VIS spectrum of the liquid-phase exfoliated MoS_2 sample.

In addition, we probed the formation of defects upon irradiation with electron beam. In the supporting video associated to Fig. S9 and available on-line at link:

https://drive.google.com/file/d/1TRUcYQzr_ejpID0dhDWB3MJ07cStHhaX/view

We found that local rearrangements occur, in particular at the edge of the flakes, due to the presence of non-saturated bonds.

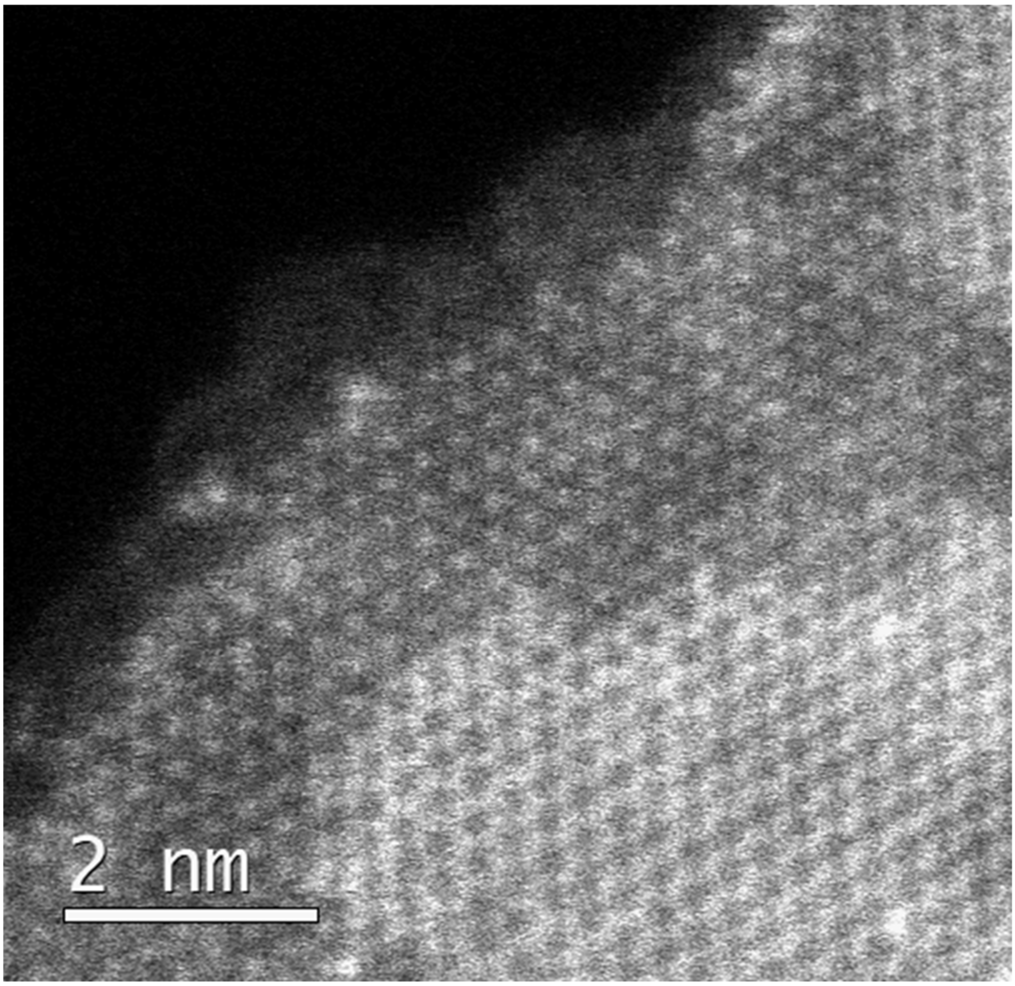

Figure S9. HR-HAADF-STEM sequence of a MoS_2 flake exfoliated with Polarclean. The number of subsequent frames in the sequence is 70. The acquisition time for each frame is about 1s. Link for the video: https://drive.google.com/file/d/1TRUcYQzr_ejpID0dhDWB3MJ07cStHhaX/view

To assess eventual influence of liquid-phase exfoliation with Polarclean solvent on MoS_2, we performed XPS characterization. Core-level spectra of bulk and exfoliated MoS_2 are shown in Fig. S10. The Mo-3d core levels are split in J=5/2 and 3/2 components shifted by 3.1 eV. Specifically, XPS measurements indicate that Mo-3d core levels have two different contributions arising from pristine (fully coordinated atoms) and defective MoS_2 (sulphur vacancies, related to vacancy neighbouring atoms) with a binding energy (BE) of 229.8 and 229.2 eV for the J=5/2 component, respectively, in agreement with previous works on MoS_2-based systems [12]. Actually, defects introduce a minority component at lower BE, due to a redistribution of charge. Specifically, the charge localized on the more electronegative sulphur atom, once it is desorbed, is redistributed on the first neighbouring atoms, so as to enhance the Coulomb screening effect [13-14]. Notably, we note in Mo-3d spectra the absence of MoO_3-derived spectral features, which should appear at a BE of 232.4 eV for the J=5/2 component [15]. Therefore, we can conclude that Polarclean does not act as an oxidation agent for the MoS_2 nanosheets and, correspondingly, Polarclean-assisted liquid-phase exfoliation process does not favor the oxidation of MoS_2 flakes.

Concerning the S-2p core levels, they are split in J=1/2 and 3/2 components shifted by 1.2 eV. Two well-distinct contributions associated to pristine and defective MoS_2 are observed at a BE of 162.5 and 161.5 eV for the J=3/2 component, respectively, as in previous reports [16-19]. No trace of sulphur-oxide phases is found, contrarily to the case of WS_2 reported in the main text in Fig. 5, for which we observed spectral contributions from both SO_4 and SO_3 phases at a BE of 168.4 and 166.7 for the J=3/2 component, respectively, as in previous reports [16-19].

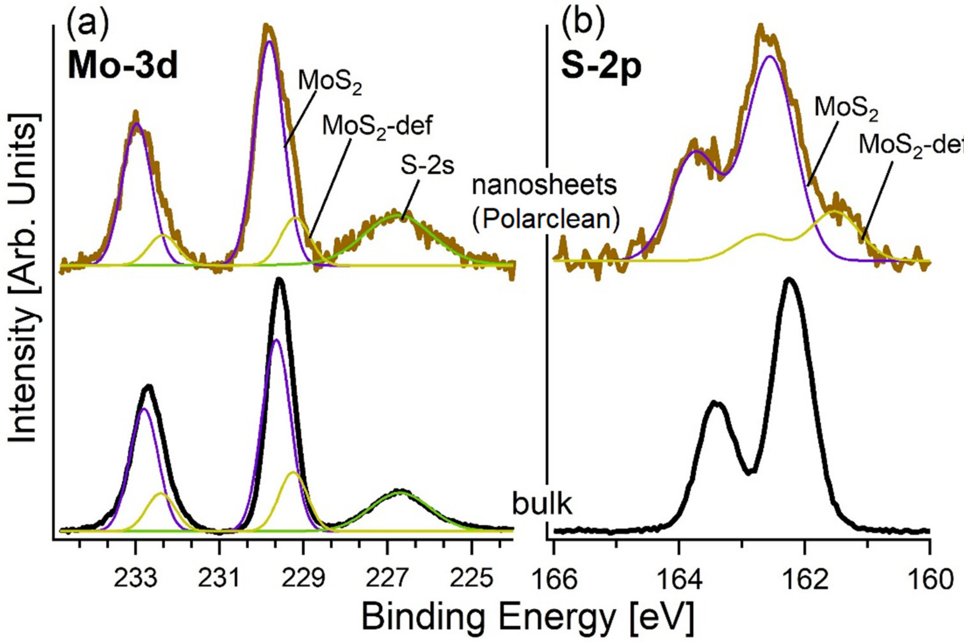

Figure S10. (a) Mo-3d and (b) S-2p core-level XPS spectra of bulk and nanosheets of MoS_2 exfoliated in Polarclean. The component at a BE of 226.7 eV in panels (a) is related to the overlapping S-2s core level. The photon energy is 1486.6 eV (Al K_α) and the spectra are normalized to the maximum.

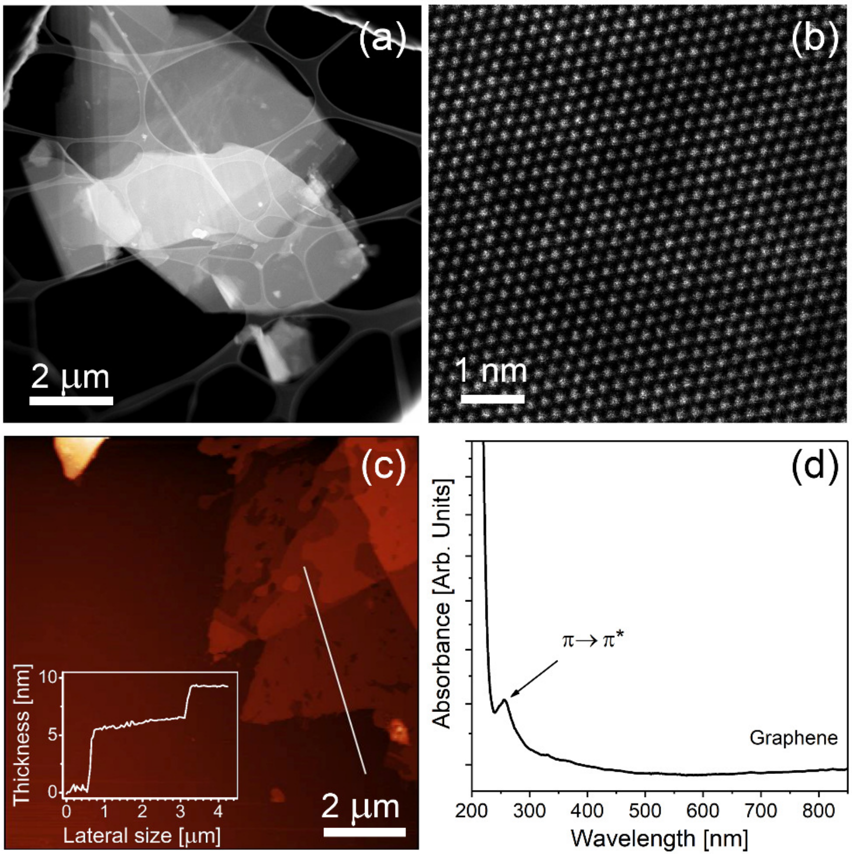

Figure S11. (a) Low-magnification HAADF-STEM image of a graphene flake exfoliated with Polarclean; (b) High magnification HAADF-STEM image of one edge of exfoliated graphene flake; (c) Representative AFM image of a graphene flake exfoliated with Polarclean; (d) UV-VIS spectrum of the dispersion of graphene flakes exfoliated with Polarclean

As for the cases of liquid-phase exfoliation of WS_2 and MoS_2, we characterized the morphological and physicochemical properties of nanosheets of graphene produced by Polarclean-assisted liquid-phase exfoliation. Specifically, HAADF–STEM images shown in Fig. S11, panels a-b, confirm the presence of large-area graphene flakes, with a lateral size exceeding 10 μm, with outstanding crystalline quality and minimized amount of defects. Typically, defects in graphene structure can be imaged by using HR-TEM [20], but we were unable to detect defects in HR-TEM images of our liquid-phase exfoliated graphene nanosheets, consistently with the particularly low value (0.07±0.01) of the I(D)/I(G) ratio measured by Raman spectroscopy (see Fig. 7 in main text and its related discussion). The inspection of a characteristic AFM image (Fig. S11c) confirms the occurrence of large-area graphene flakes with a terraced structure, as evidenced by the analysis of the height profile. Lastly, we measured the absorption spectrum of Polarclean-exfoliated graphene dispersion (Fig. S11d). The observation of the $\pi \rightarrow \pi^*$ transition [21-22] around 265 nm ensures the absence of modifications in the band structure of exfoliated graphene nanosheets.

S5. Comparison of Raman spectra of liquid-phase exfoliated graphene with different solvents

Figure S12 and Table S2 report a detailed comparison in terms of I(D)/I(G) ratio and density of defects n_D for graphene obtained with LPE using Polarclean and other solvents: NMP [23-24], cyrene [23], IPA [25], acetone/water [26], DMF [27], ethanol/water [28], TEA [29], aqueous solution of urea [30]. Precisely, the estimation of the density of defects, according to the method presented in Ref. [31], also consider the laser wavelength λ_L used for Raman measurements, following:

$$n_D\ (\mathrm{cm}^{-2}) = \frac{(1.8 \pm 0.5) \times 10^{22}}{\lambda_L^4}\left(\frac{I_D}{I_G}\right)$$

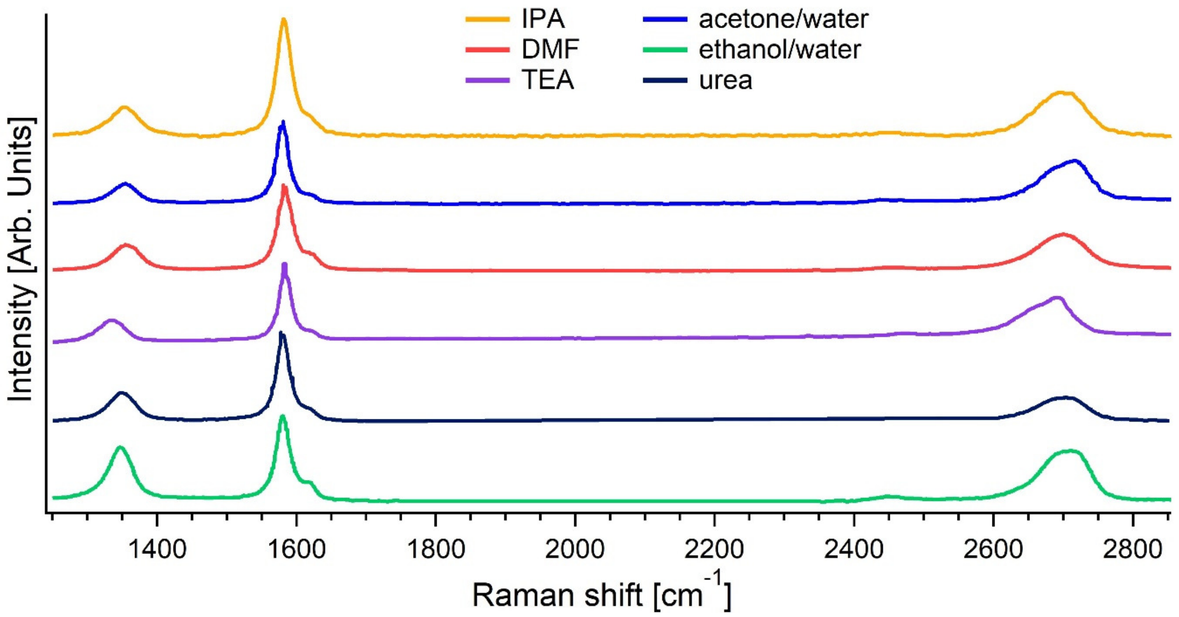

Figure S12. Comparison of Raman spectra of graphene obtained by LPE, using the following solvents: IPA (yellow curve [25]), acetone/water mixture (blue curve, data taken from Ref. [26]), DMF (red curve [27]), ethanol/water mixture (green curve, data taken from Ref. [28]), TEA (purple curve [29]) and aqueous solution of urea (blue curve, data taken from Ref. [30]).

Table S2. Comparison of I(D)/I(G) ratios and density of defects, calculated from raw data of Raman spectra of Figure 7 and Figure S12. For I(D)/I(G) of NMP, cyrene, IPA, acetone/water, DMF, ethanol/water, TEA and aqueous solution of urea, we report the value that we estimate from the analysis of raw data, together with the declared value in the related literature papers.

**Solvent used for LPE**	**Laser Wavelength [nm]**	**I(D)/I(G) ratio**	**Density of defects [defects/cm^{-2}]**
Polarclean [This Work]	633	0.07 ± 0.01	$(8 \pm 2) \cdot 10^{9}$
NMP [24]	633	0.52 ± 0.01 (~ 0.4)	$(6 \pm 2) \cdot 10^{10}$
TEA [29]	633	0.51 ± 0.01	$(6 \pm 2) \cdot 10^{10}$

Urea [30]	633	0.64 ± 0.05 (~ 0.26)	$(7 \pm 2) \cdot 10^{10}$
IPA [25]	532	0.46 ± 0.01 (~ 0.24)	$(1.0 \pm 0.3) \cdot 10^{11}$
acetone/water [26]	514	0.14 ± 0.01 (~ 0.25)	$(4 \pm 1) \cdot 10^{10}$
DMF [27]	532	0.35 ± 0.01 (~ 0.33)	$(9 \pm 3) \cdot 10^{10}$
ethanol/water [28]	532	1.01 ± 0.01 (0.15-0.45)	$(2.6 \pm 0.7) \cdot 10^{11}$

REFERENCES

1. Lyklema, J., The surface tension of pure liquids: Thermodynamic components and corresponding states. *Colloids & Surfaces, A* **1999**, *156*, 413-421.
2. Coleman, J. N.; Lotya, M.; O'Neill, A.; Bergin, S. D.; King, P. J.; Khan, U.; Young, K.; Gaucher, A.; De, S.; Smith, R. J.; Shvets, I. V.; Arora, S. K.; Stanton, G.; Kim, H.-Y.; Lee, K.; Kim, G. T.; Duesberg, G. S.; Hallam, T.; Boland, J. J.; Wang, J. J.; Donegan, J. F.; Grunlan, J. C.; Moriarty, G.; Shmeliov, A.; Nicholls, R. J.; Perkins, J. M.; Grieveson, E. M.; Theuwissen, K.; McComb, D. W.; Nellist, P. D.; Nicolosi, V., Two-Dimensional Nanosheets Produced by Liquid Exfoliation of Layered Materials. *Science* **2011**, *331*, 568-571.
3. Hernandez, Y.; Nicolosi, V.; Lotya, M.; Blighe, F. M.; Sun, Z.; De, S.; McGovern, I. T.; Holland, B.; Byrne, M.; Gun'ko, Y. K.; Boland, J. J.; Niraj, P.; Duesberg, G.; Krishnamurthy, S.; Goodhue, R.; Hutchison, J.; Scardaci, V.; Ferrari, A. C.; Coleman, J. N., High-yield production of graphene by liquid-phase exfoliation of graphite. *Nat. Nanotechnol.* **2008**, *3*, 563-568.
4. Hernandez, Y.; Lotya, M.; Rickard, D.; Bergin, S. D.; Coleman, J. N., Measurement of Multicomponent Solubility Parameters for Graphene Facilitates Solvent Discovery. *Langmuir* **2009**, *26*, 3208-3213.
5. Texter, J., Graphene dispersions. *Curr. Opin. Colloid Interface Sci.* **2014**, *19*, 163-174.
6. Shen, J.; He, Y.; Wu, J.; Gao, C.; Keyshar, K.; Zhang, X.; Yang, Y.; Ye, M.; Vajtai, R.; Lou, J., Liquid phase exfoliation of two-dimensional materials by directly probing and matching surface tension components. *Nano Lett.* **2015**, *15*, 5449-5454.
7. Wang, M.; Xu, X.; Ge, Y.; Dong, P.; Baines, R.; Ajayan, P. M.; Ye, M.; Shen, J., Surface tension components ratio: an efficient parameter for direct liquid phase exfoliation. *ACS Appl. Mater. Interfaces* **2017**, *9*, 9168-9175.
8. Backes, C.; Szydlowska, B. M.; Harvey, A.; Yuan, S.; Vega-Mayoral, V.; Davies, B. R.; Zhao, P.; Hanlon, D.; Santos, E. J. G.; Katsnelson, M. I.; Blau, W. J.; Gadermaier, C.; Coleman, J. N., Production of Highly

Monolayer Enriched Dispersions of Liquid-Exfoliated Nanosheets by Liquid Cascade Centrifugation. *ACS Nano* **2016**, *10*, 1589.
9. Pagona, G.; Bittencourt, C.; Arenal, R.; Tagmatarchis, N., Exfoliated semiconducting pure 2H-MoS_2 and 2H-WS_2 assisted by chlorosulfonic acid. *Chem. Commun.* **2015**, *51*, 12950-12953.
10. Mak, K. F.; Lee, C.; Hone, J.; Shan, J.; Heinz, T. F., Atomically thin MoS_2: A new direct-gap semiconductor. *Phys. Rev. Lett.* **2010**, *105*, 136805.
11. Forsberg, V.; Zhang, R.; Bäckström, J.; Dahlström, C.; Andres, B.; Norgren, M.; Andersson, M.; Hummelgård, M.; Olin, H., Exfoliated MoS_2 in water without additives. *PLoS One* **2016**, *11*.
12. Donarelli, M.; Bisti, F.; Perrozzi, F.; Ottaviano, L., Tunable sulfur desorption in exfoliated MoS2 by means of thermal annealing in ultra-high vacuum. *Chem. Phys. Lett.* **2013**, *588*, 198-202.
13. McIntyre, N. S.; Spevack, P. A.; Beamson, G.; Briggs, D., Effects of argon ion bombardment on basal plane and polycrystalline MoS_2. *Surface Science* **1990**, *237*, L390-L397.
14. Baker, M. A.; Gilmore, R.; Lenardi, C.; Gissler, W., XPS investigation of preferential sputtering of S from MoS2 and determination of MoSx stoichiometry from Mo and S peak positions. *Applied Surface Science* **1999**, *150*, 255-262.
15. Alov, N. V., XPS study of MoO_3 and WO_3 oxide surface modification by low-energy Ar^+ ion bombardment. *Phys. Status Solidi C* **2015**, *12*, 263-266.
16. Di Paola, A.; Palmisano, L.; Venezia, A.; Augugliaro, V., Coupled semiconductor systems for photocatalysis. Preparation and characterization of polycrystalline mixed WO_3/WS_2 powders. *J. Phys. Chem. B* **1999**, *103*, 8236-8244.
17. Wong, K.; Lu, X.; Cotter, J.; Eadie, D.; Wong, P.; Mitchell, K., Surface and friction characterization of MoS_2 and WS_2 third body thin films under simulated wheel/rail rolling–sliding contact. *Wear* **2008**, *264*, 526-534.
18. Donarelli, M.; Prezioso, S.; Perrozzi, F.; Bisti, F.; Nardone, M.; Giancaterini, L.; Cantalini, C.; Ottaviano, L., Response to NO_2 and other gases of resistive chemically exfoliated MoS2-based gas sensors. *Sensors and Actuators B: Chemical* **2015**, *207*, 602-613.
19. Shpak, A.; Korduban, A.; Kulikov, L.; Kryshchuk, T.; Konig, N.; Kandyba, V., XPS studies of the surface of nanocrystalline tungsten disulfide. *J. Electron Spectrosc. Relat. Phenom.* **2010**, *181*, 234-238.
20. Robertson, A. W.; Warner, J. H., Atomic resolution imaging of graphene by transmission electron microscopy. *Nanoscale* **2013**, *5*, 4079-4093.
21. Goodwin, D. G.; Adeleye, A. S.; Sung, L.; Ho, K. T.; Burgess, R. M.; Petersen, E. J., Detection and Quantification of Graphene-Family Nanomaterials in the Environment. *Environ. Sci. Technol.* **2018**, *52*, 4491-4513.
22. Grüneis, A.; Attaccalite, C.; Wirtz, L.; Shiozawa, H.; Saito, R.; Pichler, T.; Rubio, A., Tight-binding description of the quasiparticle dispersion of graphite and few-layer graphene. *Phys. Rev. B* **2008**, *78*, 205425.
23. Salavagione, H. J.; Sherwood, J.; Budarin, V.; Ellis, G.; Clark, J.; Shuttleworth, P., Identification of high performance solvents for the sustainable processing of graphene. *Green Chem.* **2017**, *19*, 2550-2560.
24. Khan, U.; Porwal, H.; O'Neill, A.; Nawaz, K.; May, P.; Coleman, J. N., Solvent-exfoliated graphene at extremely high concentration. *Langmuir* **2011**, *27*, 9077-9082.
25. Çelik, Y.; Flahaut, E.; Suvacı, E., A comparative study on few-layer graphene production by exfoliation of different starting materials in a low boiling point solvent. *FlatChem* **2017**, *1*, 74-88.
26. Yi, M.; Shen, Z.; Zhang, X.; Ma, S., Achieving concentrated graphene dispersions in water/acetone mixtures by the strategy of tailoring Hansen solubility parameters. *J. Phys. D: Appl. Phys.* **2012**, *46*, 025301.
27. Zhang, R.; Zhang, B.; Sun, S., Preparation of high-quality graphene with a large-size by sonication-free liquid-phase exfoliation of graphite with a new mechanism. *RSC Adv.* **2015**, *5*, 44783-44791.
28. Capasso, A.; Castillo, A. D. R.; Sun, H.; Ansaldo, A.; Pellegrini, V.; Bonaccorso, F., Ink-jet printing of graphene for flexible electronics: an environmentally-friendly approach. *Solid State Commun.* **2015**, *224*, 53-63.
29. Chen, H.; Liu, B.; Yang, Q.; Wang, S.; Liu, W.; Zheng, X.; Liu, Z.; Liu, L.; Xiong, C., Facile one-step exfoliation of large-size 2D materials via simply shearing in triethanolamine. *Mater. Lett.* **2017**, *199*, 124-127.

30. He, P.; Zhou, C.; Tian, S.; Sun, J.; Yang, S.; Ding, G.; Xie, X.; Jiang, M., Urea-assisted aqueous exfoliation of graphite for obtaining high-quality graphene. *Chem. Commun.* **2015**, *51*, 4651-4654.
31. Cançado, L. G.; Jorio, A.; Ferreira, E. H. M.; Stavale, F.; Achete, C. A.; Capaz, R. B.; Moutinho, M. V. O.; Lombardo, A.; Kulmala, T. S.; Ferrari, A. C., Quantifying Defects in Graphene via Raman Spectroscopy at Different Excitation Energies. *Nano Lett.* **2011**, *11*, 3190–3196.
